# Application of a Neural Network Whole Transcriptome–Based Pan-Cancer Method for Diagnosis of Primary and Metastatic Cancers

**DOI:** 10.1001/jamanetworkopen.2019.2597

**Published:** 2019-04-26

**Authors:** Jasleen K. Grewal, Basile Tessier-Cloutier, Martin Jones, Sitanshu Gakkhar, Yussanne Ma, Richard Moore, Andrew J. Mungall, Yongjun Zhao, Michael D. Taylor, Karen Gelmon, Howard Lim, Daniel Renouf, Janessa Laskin, Marco Marra, Stephen Yip, Steven J. M. Jones

**Affiliations:** 1 Canada’s Michael Smith Genome Sciences Centre, Vancouver, British Columbia, Canada; 2 Department of Pathology and Laboratory Medicine, University of British Columbia, Vancouver, British Columbia, Canada; 3 Division of Neurosurgery, Hospital for Sick Children, Toronto, Ontario, Canada; 4 Division of Medical Oncology, BC Cancer, Vancouver, British Columbia, Canada; 5 Department of Medical Genetics, University of British Columbia, Vancouver, British Columbia, Canada

## Abstract

**Importance:**

A molecular diagnostic method that incorporates information about the transcriptional status of all genes across multiple tissue types can strengthen confidence in cancer diagnosis.

**Objective:**

To determine the practical use of a whole transcriptome–based pan-cancer method in diagnosing primary and metastatic cancers and resolving complex diagnoses.

**Design, Setting, and Participants:**

This cross-sectional diagnostic study assessed Supervised Cancer Origin Prediction Using Expression (SCOPE), a machine learning method using whole-transcriptome RNA sequencing data. Training was performed on publicly available primary cancer data sets, including The Cancer Genome Atlas. Testing was performed retrospectively on untreated primary cancers and treated metastases from volunteer adult patients at BC Cancer in Vancouver, British Columbia, from January 1, 2013, to March 31, 2016, and testing spanned 10 822 samples and 66 output classes representing untreated primary cancers (n = 40) and adjacent normal tissues (n = 26). SCOPE’s performance was demonstrated on 211 untreated primary mesothelioma cancers and 201 treatment-resistant metastatic cancers. Finally, SCOPE was used to identify the putative site of origin in 15 cases with initial presentation as cancers with unknown primary of origin.

**Results:**

A total of 10 688 adult patient samples representing 40 untreated primary tumor types and 26 adjacent-normal tissues were used for training. Demographic data were not available for all data sets. Among the training data set, 5157 of 10 244 (50.3%) were male and the mean (SD) age was 58.9 (14.5) years. Testing was performed on 211 patients with untreated primary mesothelioma (173 [82.0%] male; mean [SD] age, 64.5 [11.3] years); 201 patients with treatment-resistant cancers (141 [70.1%] female; mean [SD] age, 55.6 [12.9] years); and 15 patients with cancers of unknown primary of origin; among the treatment-resistant cancers, 168 were metastatic, and 33 were the primary presentation. An accuracy rate of 99% was obtained for primary epithelioid mesotheliomas tested (125 of 126). The remaining 85 mesotheliomas had a mixed etiology (sarcomatoid mesotheliomas) and were correctly identified as a mixture of their primary components, with potential implications in resolving subtypes and incidences of mixed histology. SCOPE achieved an overall mean (SD) accuracy rate of 86% (11%) and *F*_1_ score of 0.79 (0.12) on the 201 treatment-resistant cancers and matched 12 of 15 of the putative diagnoses for cancers with indeterminate diagnosis from conventional pathology.

**Conclusions and Relevance:**

These results suggest that machine learning approaches incorporating multiple tumor profiles can more accurately identify the cancerous state and discriminate it from normal cells. SCOPE uses the whole transcriptomes from normal and tumor tissues, and results of this study suggest that it performs well for rare cancer types, primary cancers, treatment-resistant metastatic cancers, and cancers of unknown primary of origin. Genes most relevant in SCOPE’s decision making were examined, and several are known biological markers of respective cancers. SCOPE may be applied as an orthogonal diagnostic method in cases where the site of origin of a cancer is unknown, or when standard pathology assessment is inconclusive.

## Key Points

**Question:**

What is the practical use of a computational method trained to classify cancer types using the whole transcriptome?

**Findings:**

For this cross-sectional diagnostic analysis, a set of neural networks was trained using the whole transcriptomes of normal and tumor tissues; the resultant classifier had a 99% accuracy rate for identifying primary cancers in an independent cohort, showed stable performance in treatment-resistant metastases, and identified 12 of 15 putative primary tumors for cancers with unknown site of origin.

**Meaning:**

According to results of this study, machine learning–based cancer classifiers that use the whole transcriptome to automatically learn tissue- and tumor-specific gene markers in an unsupervised manner may resolve cases refractory to routine pathology diagnosis.

## Teaser

This cross-sectional diagnostic study evaluates the accuracy of a machine learning method that uses the whole transcriptome to identify gene markers in primary and metastatic tumors.

## Introduction

Identification of the site of origin of a tumor in a patient is currently used to guide cancer treatment. It also informs any subsequent analysis through alignment with relevant tumor literature and expected molecular background. Currently, established pathology approaches are used for cancer diagnosis and are considered the criterion standard. These approaches use morphology and histochemistry to provide a diagnosis that also determines eligibility for drug regimens and clinical trials. Modern pathology is a process of sequential exclusion and prioritization across candidate diagnoses, but an exhaustive search is rendered impossible by limited tissue and diagnostic stains.

The efficiency of cancer diagnosis by pathologists may be improved if an automated method can be developed to approach this task with some knowledge of cancer biology, similar to a pathologist. A machine learning method trained across diverse tumors and normal tissues can learn what characterizes each cancer, rather than its tissue site. Training on high-resolution molecular data will allow it to discover tissue-specific and tumor-specific biological patterns from the whole transcriptome.

The use of gene-expression data has outperformed traditional pathology workflows for cancer diagnosis in several landmark studies.^1,2,3,4^ Studies have also shown that transcriptome-wide profiling offers greater information about tumors than microarrays,^5,6^ with practical use in precision oncology.^7,8^ We can therefore use high-resolution transcriptomic data as an orthogonal approach to improve diagnostic accuracy in many cancers.^9,10^ Although analyzing such high-dimensional data within a diagnostic workflow is not manually feasible, machine learning methods can be trained to do so instead.

We have developed and validated Supervised Cancer Origin Prediction Using Expression (SCOPE), a set of neural networks that use the whole transcriptome to identify the closest match for a tumor from among 40 cancer types and 26 normal tissues. We account for the influence of differentiation and biopsy site by including normal tissues (classes) from The Cancer Genome Atlas (TCGA) in our training data set.^11^ We determine genes weighted heavily for decision making and demonstrate that SCOPE is able to prioritize genes relevant to each class without any prior information. Our method takes the reads per kilobase of transcript per million (RPKM) values of 17 688 genes to predict a tumor type in less than 2 minutes per sample on a CPU machine with 32 GB RAM, and can be extended as new data become available.

### Diagnostic Challenges in Pathology

Pathology protocols for cancer diagnosis work best when the tissue specimens display high-quality and recognizable histologic features in a substantial number of cells. Generic histologic features alone are often not sufficient to determine the subtype of a tumor; hence, the confirmation of cell of origin, typically via immunohistochemical analysis, remains the bedrock of modern pathology practice.^12^ Therefore, diagnosis can become a challenging task of tiered, single-plex immunohistochemical analyses for lineage-specific proteins, iteratively evaluating the next-likely diagnostic candidates. Limited tissue availability and a limited list of unambiguous immunohistochemical antibodies restrict the extent of validation workups. Interobserver variability and sample-related issues further confound pathology diagnoses.^13^

### Diagnostic Confounders

Misdiagnosis rates for metastases in clinical practice can range between 45% and 94% in the event of challenging presentation (eg, suboptimal sample quality, histologic similarity between tissues, poor differentiation).^14^ This is concerning because metastases can form up to 60% of distant recurrences and cause upward of 90% of cancer-associated deaths for cancers detected in the gastrointestinal tract and across certain gynecologic cancers.^13,15,16^ Biomarker conversion in metastases can confound diagnosis using immunohistochemistry or biomarker-based assays.^5^ The site of biopsy is another confounder, particularly in case of the liver.^17^ Previous work using expression microarrays has indicated that the microenvironment can contribute to the enrichment of hepatic genes’ expression in liver metastases, confounding an accurate diagnosis.^18^ These issues are magnified in cancers of unknown primary of origin (CUPs), where developing specific diagnostic protocols remains a challenge for pathology.^3,9,10^

### Application of Machine Learning in Cancer Diagnosis

The first application of machine learning in molecular cancer diagnosis discriminated small, round blue cell tumors using microarray data.^19^ Khan et al^19^ observed that difficulties in interpreting morphologic features and reverse transcriptase polymerase chain reaction results were easily overcome when using neural networks to classify these cancers. Other machine learning algorithms have been applied to larger cohorts of cancer microarrays.^3,20,21^ Within these studies, samples are separated into training and test sets, and representative genes (features) are selected to maximize accuracy on the test set.

Including rare cancer types and providing a refined diagnosis remain challenges for current computational diagnostic methods. To optimize training, rare cancer types are often excluded, and geographically proximal cancers are merged. This inevitably leads to loss of granularity and limited scope in the application of the models trained.^3,20^ Performance is evaluated on the test set, which can either be held out from the initial cohort or, preferably (but rarely), a cohort of samples generated and processed at different centers.^3,20,21^

### RNA Sequencing: Incorporating High-Resolution Sequencing Data in Diagnostic Methods

RNA sequencing has largely replaced microarrays for transcriptome-wide profiling. However, the current repertoire of diagnostic methods does not draw on the high dynamic range and comprehensive coverage provided by RNA sequencing.^8,22^ Large-scale sequencing projects (TCGA,^23^ International Cancer Genome Consortium [ICGC]^24^) have amassed RNA sequencing data from approximately 10 000 patients with untreated primary cancers. The size and diversity of this data set provides unprecedented opportunity to apply machine learning approaches to improve the classification of all cancer types. With the availability of high-performance computing systems, it is now possible to train models using information about the transcriptional status of all genes.

## Methods

We trained an ensemble of neural networks using four-fifths of the TCGA cohort of primary cancers and normal tissues. We evaluated the method on the remaining one-fifth of the TCGA cohort and tested for robustness on the Genentech cohort of primary mesotheliomas.^25^ We demonstrated its application on extensively pretreated metastatic lesions and CUPs. A linear classifier using analysis of variance (ANOVA)–selected features^26^ was used to establish the baseline performance for the classifier in the metastatic cohort (eAppendix 1 in the Supplement). The study was conducted on retrospective data from January 1, 2013, to March 31, 2016, and no follow-up was required. The Personalized OncoGenomics project at BC Cancer is approved by the University of British Columbia BC Cancer Agency Research Ethics Board. Eligible patients living in British Columbia were referred to the program by their treating oncologist. Written informed consent for the selected patient population was obtained between January 1, 2013, and March 31, 2016. This study followed the Standards for Reporting of Diagnostic Accuracy (STARD) reporting guidelines for diagnostic analyses.

### Data Preparation

#### Training Data

Multiplatform RNA sequencing data were obtained from TCGA, the National Cancer Institute’s non-Hodgkin lymphoma data set, and in-house data sets of secondary glioblastoma, adult medulloblastoma, and follicular lymphoma, available for research and development within our institution by agreement. Colon and rectal adenocarcinomas were combined into a single cohort (COADREAD) based on TCGA consortium findings.^23^ This resulted in 10 822 samples spanning 40 different tumor types and 26 adjacent normal classes, with each sample represented by 17 688 distinct gene reads per kilobase of transcript per million (RPKM) values (eAppendix 2 in the Supplement).

#### Test Data

Testing was performed retrospectively. An independent set of 211 adult primary untreated mesothelioma cancers was obtained from the Genentech mesothelioma cohort.^25^ One hundred twenty-six of these samples are classic epithelioid mesotheliomas, whereas 85 are sarcomatoid variants. Because the training set of mesotheliomas was histologically classic epithelioid mesotheliomas, testing was performed as follows: For the epithelioid mesotheliomas, we tested whether the classification was exclusively for mesothelioma. For the biphasic and sarcomatoid variants, we tested whether the classification was split between sarcomas and mesotheliomas, as would be expected based on mixed histology of the samples. Test sets for adult metastatic disease and 15 CUPs were obtained retrospectively from the Personalized OncoGenomics study at BC Cancer.^7^ Biopsy specimens of 168 of the 201 metastases were obtained from their site of metastasis (24 cancer types), and the remaining 33 from their site of origin (12 cancer types) (eAppendix 3, eTable 1, and eTable 2 in the Supplement).

### Data Preprocessing and Model Selection

For the initial selection of the optimal classification algorithm, gene RPKMs were used as input (eAppendix 4 and eAppendix 5 in the Supplement). Support vector machines, random forests, extra trees, and a fully connected neural network were compared. Five-cross validation with grid search was used to identify the best parameters for each of these algorithms. The trained models were subsequently tested on the one-fifth held-out set.

Because the other ensemble models (random forest, extra trees) had near-equivalent 5-cross validation results with the neural network during training, we evaluated the utility of extending the neural network model. An ensemble was developed by training multiple neural networks with different linear transformations of the data. The resultant classifier (SCOPE) contained 5 neural networks. For 1 of these neural networks, we generated synthetically generated samples to expand the rarer classes during training (Synthetic Minority Oversampling Technique [SMOTE]^27^). The differences in the 5 networks are described in detail in eAppendix 1 and eTable 3 in the Supplement.

### Assessing Use of Feature Selection

After selection of the optimal algorithm as described, we tested the practical use of feature selection in improving classification performance. Guided by previous work,^28^ we used pairwise ANOVA of log-transformed training data to identify a subset of 3000 genes that are statistically significant at discriminating the training classes. We also trained a classifier using the Catalogue of Somatic Mutations in Cancer’s list of 552 genes harboring somatic mutations.^29^ Neural network architectures optimal for each input space were identified using grid search across parameters, and trained with 5-cross validation for comparison.

### Analysis of Results

The confidence score for a prediction was calculated as follows: Each neural network in the ensemble generated prediction probabilities between 0 and 1 for each class, all of which sum to 1. The class with the highest probability was considered the top-voted class for that neural network. The class top-voted most frequently across the ensemble was identified. The confidence score was then calculated as the mean of those top-voting scores.

Weight analysis of neural network connections was used to identify genes that were most important for predicting each class (eAppendix 6 in the Supplement).

### Statistical Analysis

Precision, recall, and *F*_1_ score were used to evaluate models and demonstrate their performance. Aggregate precision and *F*_1_ scores, where reported in text, are accompanied by 95% CIs. Precision is defined as (true-positives)/(true-positives + false-positives), and intuitively represents the classifier’s ability to distinguish between positive and negative cases. Recall is defined as (true-positives)/(true-positives + false-negatives), and intuitively represents the classifier’s ability to correctly identify all positive cases. The *F*_1_ score is the harmonic mean of the precision and recall. These metrics are calculated for each individual class, and the mean reported as the cohort metric. Accuracy is reported as (true-positives + true-negatives)/(total cases), and is calculated for the entire cohort.

A paired χ^2^ test for association between prediction accuracy and tumor content was performed on the metastatic test cohort, with the null hypothesis being, “the classification accuracy of SCOPE is independent of tumor content.” Tumor content was determined by pathology analysis. A paired *t* test was used to test the association between prediction accuracy and confidence score (null hypothesis: no correlation exists between prediction accuracy and confidence score). The level of significance was 2-sided *P* = .05 for all tests of association. Pearson correlation was used to evaluate association between class-specific accuracy and training class size. Statistical tests were conducted using the base statistics package available in R (R version 3.5.0; RStudio API version 1.1.442; R Project for Statistical Computing).

## Results

**Figure 1.**
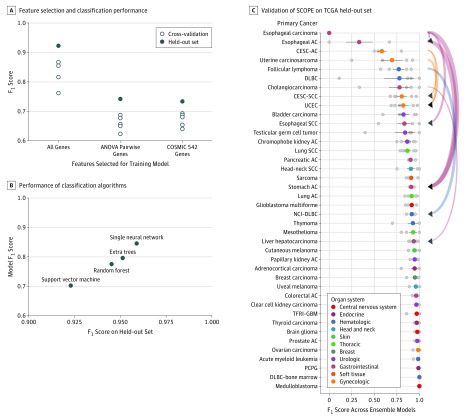
Results From Algorithm and Feature Selection Experiments and Performance of Supervised Cancer Origin Prediction Using Expression (SCOPE) on The Cancer Genome Atlas (TCGA) Held-Out Set A, Feature selection does not improve pan-cancer classification, with the single neural network having a higher performance than neural networks trained on biologically or statistically relevant gene subsets. B, Performance of single neural network on held-out set is higher than other algorithms. C, Validation of SCOPE on the TCGA held-out set shows high discriminatory power among most cancer types. Incorrect predictions for more than 10% of samples belonging to a given cancer types are shown by curved directed edges. Curve width shows relative fraction of samples in misprediction set. Mispredictions occur among cancer type with same organ system of origin. Colored points with bars represent mean *F*_1_ score and SD spread for the corresponding category. Individual gray points in each category indicate the held-out performance of a single neural network part of the ensemble (n = 5). AC indicates adenocarcinoma; CESC-AC, cervical/endocervical adenocarcinoma, CESC-SCC, cervical/endocervical squamous cell carcinoma; DLBC, diffuse large B-cell lymphoma; NCI-DLBC, National Cancer Institute’s cohort of DLBC; PCPG, pheochoromocytoma/paraganglioma; SCC, squamous cell carcinoma; TFRI-GBM, Terry Fox Research Institute’s cohort of non–cell line glioblastoma; UCEC, uterine corpus endometrial carcinoma.

A total of 10 688 adult patient samples representing 40 untreated primary tumor types and 26 adjacent-normal tissues were used for training. Demographic data were not available for all data sets. Among the training data set, 5157 of 10 244 (50.3%) were male and the mean (SD) age was 58.9 (14.5) years. Testing was performed on 211 patients with untreated primary mesothelioma (173 [82.0%] male; mean [SD] age, 64.5 [11.3] years); 201 patients with treatment-resistant cancers (141 [70.1%] female; mean [SD] age, 55.6 [12.9] years); and 15 patients with cancers of unknown primary of origin; among the treatment-resistant cancers, 168 were metastatic, and 33 were the primary presentation. In our study, SCOPE achieved 97% accuracy and a macro *F*_1_ score of 0.92 on the 2780 cases in the TCGA held-out set. We found that the whole transcriptome had improved performance over the Catalogue of Somatic Mutations in Cancer cancer gene set and ANOVA-selected genes (Figure 1A; eFigure 1 and eAppendix 7 in the Supplement). The single neural network outperformed other machine learning algorithms (Figure 1B; eFigure 2 in the Supplement). For 46 of 66 classes, 80% to 100% of the samples in each class were correctly classified (Figure 1C; eFigure 3 in the Supplement). We found that 7 classes were refractory to appropriate classification, among which 3 were cancer types (esophageal carcinomas and adenocarcinomas and cervical cancers), and all 7 had fewer than 50 training examples (class size range, 3-50). On closer investigation of the 5 neural networks in the ensemble, we found that the neural network trained with SMOTE-supplemented training examples showed improved performance on smaller classes compared with the other 4 (eFigure 4 in the Supplement).

### Association of Classification Anomalies and Biological Similarities in Held-Out Set

Among the poor-performing classes in the TCGA held-out set, certain patterns were evident. The 3 kidney adjacent-normal classes (KICH, KIRC, KIRP) had significant cross-calling, which was as expected because all 3 represent healthy kidney tissue (eFigure 3B in the Supplement).

Esophageal carcinomas and adenocarcinomas were often misclassified as stomach adenocarcinomas (Figure 1C). For cervical cancers, which can be squamous, adenosquamous, and adenocarcinomas,^30^ subtypes were also challenging to distinguish by SCOPE. We found these trends were replicated in unsupervised clustering of the RNA sequencing data, suggesting biological rationale for the same (eFigure 5 and eFigure 6 in the Supplement).

As further evidence, we observed other molecular patterns previously noted in literature in our results. The endometrium is a common site of occurrence for uterine carcinosarcomas, and an endometrioid carcinomalike profile is a well-documented molecular subtype of uterine carcinosarcomas. We found that uterine carcinosarcoma was frequently misclassified as uterine corpus endometrial carcinoma. The Cancer Genome Atlas analysis has found that a majority of uterine carcinosarcoma samples had serouslike endometrial carcinoma precursors.^16^ This cross-calling was also observed by another group using this data set for classification.^21^

### Prioritization of Known Diagnostic Gene Features Without Prior Knowledge

Manual review showed that high-importance genes for a given class were biologically relevant to the corresponding cancer or normal tissue type. For example, 2 kidney-specific genes, *UMOD* and *AQP2*, were exclusively associated with the adjacent normal tissues from all 3 renal cancer types in training. Known diagnostic markers for renal clear cell carcinoma, namely *CA9* and *CA12*, were associated with renal clear cell carcinoma. Important genes for testicular germline cancers, *POU5F1*, *GDF3*, and *NANOG*, are known and proposed biomarkers. High *POU5F1* (OCT4) and *NANOG* expression is associated with spermatogenesis dysregulation.^31^ Unexpectedly, in the absence of a healthy tissue class corresponding to a primary tumor type, some important genes for the cancer reflect biological characteristics of the progenitor healthy tissue, such as *DPPA3*/*5* for testicular germline cancers, and *TYR* and *MLANA* for uveal melanomas. These observations underscore the value in including adjacent normal tissues for a pan-cancer classifier. Genes associated with each cancer type are detailed in eTable 4 in the Supplement.

### External Validation on Primary Cancers

Mesothelioma is a cancer that arises in the pleura, which lines the lungs. Three main histologic categories have been defined within mesothelioma: epithelioid, sarcomatoid, and a biphasic type that presents a combination of features from the former.^32^ Subtype diagnosis in mesothelioma influences patient prognosis and disease management, but without specialized histopathologist training, there is low agreement between diagnoses.^33^ We applied SCOPE on a previously published cohort of primary, untreated mesothelioma subtypes.

### Characterizing Cancers With Mixed Histology

We obtained 99.2% accuracy (125 of 126) in identifying epithelioid mesotheliomas and biphasic-epithelioid cancers in this cohort. This is as expected, because SCOPE was trained to identify epithelioid mesotheliomas (this subtype was exclusively represented in the mesothelioma training set). Twenty-three of 29 sarcomatoid mesotheliomas (79.3%) and 55 of 56 biphasic-sarcomatoid mesotheliomas (98.2%) were predicted with split confidence between mesothelioma and sarcoma (Table 1). In addition, 4 of the remaining 6 sarcomatoid subtype samples were predicted confidently as sarcomas.

**Table 1.** Performance of SCOPE on Genentech Cohort of Primary Mesotheliomas^a^

Mesothelioma Subtype	Total Cases With Subtype, No.	Precision	Recall	*F*_1_ Score	Predicted	Predicted Cases, No.
Biphasic epithelioidlike	72	1	1	1	Epithelioid mesothelioma	72
Epithelioid	54	1	0.98	0.99	Epithelioid mesothelioma	53
Sarcomatoid	29	NA	NA	NA	Sarcomatoid mesothelioma	18
		NA	NA	NA	Epithelioid mesothelioma	5
		NA	NA	NA	Sarcoma	4
		NA	NA	NA	Other	2
Biphasic sarcomalike	56	NA	NA	NA	Epithelioid mesothelioma	38
		NA	NA	NA	Sarcomatoid mesothelioma	17
		NA	NA	NA	Other	1

Abbreviations: NA, not applicable; SCOPE, Supervised Cancer Origin Prediction Using Expression.

^a^ Data from Bueno et al,^25^ 2016.

### Providing Diagnosis for Complex Metastases

**Figure 2.**
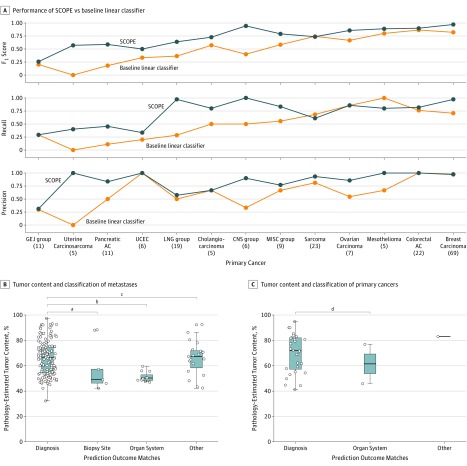
Performance of Supervised Cancer Origin Prediction Using Expression (SCOPE) on External Metastatic Cohort A, SCOPE has improved performance compared with baseline linear comparator trained from a statistically filtered feature subset. Numbers in parentheses indicate sample size. B, Two-sided *t* tests show a significant association of tumor content on general diagnosis as organ system, for biopsies sampled from site of metastasis. C, Two-sided *t* tests show no effect of tumor content on misclassification to organ system, for biopsies sampled from the cancer’s site of origin. B and C, Box plots illustrate the median (center line in box) with the lower and upper hinges indicating the 25th and 75th percentiles, respectively. The upper whisker shows the largest value at most 1.5 times the interquartile range (IQR) from the hinge, and the lower whisker shows the smallest value at most 1.5 times the IQR of the hinge. The IQR is calculated as the distance between the first and third quartiles. Data points outside these ranges are plotted as individual points. AC indicates adenocarcinoma; CESC-AC, cervical/endocervical adenocarcinoma; CNS group, lower-grade glioma, glioblastoma multiforme; GEJ group, esophageal AC, esophageal SCC, stomach AC, liver hepatocellular carcinoma, papillary kidney carcinoma; LNG group, lung AC, lung squamous cell carcinoma (SCC); MISC group, prostate AC, testicular germ cell tumor, CESC-AC, subcutaneous melanoma, diffuse large B-cell lymphoma, follicular lymphoma, thymoma, adrenocortical carcinoma; UCEC, uterine corpus endometrial carcinoma. ^a^*P* = .03. ^b^*P* < .001. ^c^*P* = .79. ^d^*P* = .70.

In an independent set of 201 posttreatment metastatic cancers, SCOPE performed well above the baseline linear classifier, achieving an overall accuracy of 86% (11%), and a mean (SD) *F*_1_ score of 0.79 (0.12) (Figure 2A; Table 2; eFigure 7, eTable 5, and eTable 6 in the Supplement). Among the 41 mispredictions, 7 (17.1%) matched the site of biopsy (for example, predicting hepatocarcinoma for a breast cancer biopsy specimen from the liver), and 13 of the 41 (31.7%) matched a cancer type with same organ system of origin instead (for example, predicting uterine carcinosarcoma as ovarian cancer, predicting stomach adenocarcinoma as esophageal adenocarcinoma). For the remaining 21 cases, no obvious explanation was found for misclassification. Because our method provided a confidence score for each prediction, we found that in the set of confident diagnoses from the ensemble (118 of 201, confidence score of ≥80%, spanning 20 cancer types) accuracy went up to 92%.

**Table 2.** Performance on the Metastatic Cohort

Diagnosed Type^a^	Total Cases, No.	Cohort Metrics^b^									Cases Predicted, No.^c^			
		TPR	FPR	TP	TN	FP	FN	Precision	Recall	*F*_1_ Score	Diagnosis	Biopsy Site	Organ System	Other
**Metastatic Site Biopsies**														
Mesothelioma	1	1.00	0.00	1	130	0	0	1.00	1.00	1.00	1	0	0	0
Colorectal AC	21	0.81	0.00	17	114	0	4	1.00	0.81	0.89	17	1	2	1
UCEC	5	0.40	0.00	2	129	0	3	1.00	0.40	0.57	2	0	1	2
Uterine carcinosarcoma	4	0.25	0.00	1	130	0	3	1.00	0.25	0.40	1	0	2	1
Breast carcinoma	65	0.97	0.03	63	68	2	2	0.97	0.97	0.97	63	1	0	1
LNG_group	14	1.00	0.01	14	117	1	0	0.93	1.00	0.97	14	0	0	0
Sarcoma	17	0.53	0.01	9	122	1	8	0.90	0.53	0.67	9	1	0	7
Ovarian carcinoma	7	0.86	0.01	6	160	1	1	0.86	0.86	0.86	6	0	0	1
Pancreatic AC	9	0.33	0.01	3	158	1	6	0.75	0.33	0.46	3	1	4	1
MISC_group	9	0.88	0.00	8	125	1	1	0.73	0.88	0.77	8	0	0	1
Cholangio-carcinoma	5	0.80	0.02	4	127	2	1	0.67	0.80	0.73	4	0	1	0
GEJ_group	11	0.29	0.01	3	151	5	8	0.31	0.29	0.26	3	3	1	4
**Primary Site Biopsies**														
CNS_group	6	1.00	0.00	6	23	0	0	1.00	1.00	1.00	6	0	0	0
Breast carcinoma	4	1.00	0.00	4	25	0	0	1.00	1.00	1.00	4	0	0	0
Colorectal AC	1	1.00	0.00	1	28	0	0	1.00	1.00	1.00	1	0	0	0
GEJ_group	1	1.00	0.00	1	28	0	0	1.00	1.00	1.00	1	0	0	0
MISC_group	2	1.00	0.00	2	27	0	0	1.00	1.00	1.00	2	0	0	0
Pancreatic AC	2	1.00	0.00	2	27	0	0	1.00	1.00	1.00	2	0	0	0
Uterine carcinosarcoma	1	1.00	0.00	1	28	0	0	1.00	1.00	1.00	1	0	0	0
Sarcoma	6	0.83	0.00	5	24	0	1	1.00	0.83	0.91	5	0	0	1
Mesothelioma	4	0.75	0.00	3	26	0	1	1.00	0.75	0.86	3	0	0	1
LNG_group	5	0.88	0.02	4	23	1	1	0.75	0.88	0.76	4	0	1	0
UCEC	1	0.00	0.00	0	29	0	1	0.00	0.00	0.00	0	0	1	0
Total	201	0.76	0.005	160	3128	19	41	0.86	0.76	0.79	160	7	13	21

Abbreviations: AC, adenocarcinoma; CESC–AC, cervical/endocervical adenocarcinoma; FN, false-negative count; FP, false-positive count; FPR, false-positive rate; SCC, squamous cell carcinoma; TN, true-negative count; TP, true-positive count; TPR, true-positive rate; UCEC, uterine corpus endometrial carcinoma.

^a^ CNS_group includes lower-grade glioma, glioblastoma multiforme. LNG_group includes lung AC, and lung SCC; GEJ_group includes esophageal AC, esophageal SCC, stomach AC, liver hepatocellularcarcinoma, and papillary kidney carcinoma. MISC_group includes prostate AC, testicular germ cell tumor, CESC-AC, subcutaneous melanoma, diffuse large B-cell lymphoma, follicular lymphoma, thymoma, and adrenocortical carcinoma.

^b^ Precision, as indicated, is equivalent to class-specific accuracy.

^c^ Cases where predicted cancer type matched pathology diagnosis (diagnosis), was the same as tissue type of the biopsy site (biopsy site), matched a cancer type with same organ system of origin (organ system), or did not match any of the above (other).

In our assessment of the metastatic cohort, we found no association between classification accuracy and tumor content (*P* = 0.59), and a weak correlation with the size of training class (Pearson correlation coefficient, 0.39). There was an association between classification accuracy and confidence score (n = 201; *P* < .001). These observations are evident in eFigure 8 in the Supplement. In biopsies from sites of metastasis (n = 168), an association was found between low tumor content and the diagnosis of another cancer type with the same organ system of origin (Figure 2B and Table 2). This association was not found in primary cancer biopsies (Figure 2C and Table 2).

### Identification of Putative Primary Tumor Type for Cancers of Unknown Primary

**Figure 3.**
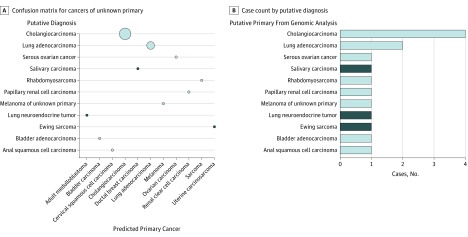
Supervised Cancer Origin Prediction Using Expression (SCOPE) Prediction and Putative Primary for Cancers of Unknown Primary Site A, Confusion matrix of predictions for primaries of unknown origin. The size of the circles represents relative number of samples in each category. B, Case count for cancers of unknown primary of origin by category. Correct predictions are shown in light blue; incorrect predictions, dark blue.

We retrospectively predicted the cancer type for 15 cancers where the primary site of origin was unknown after initial pathology assessment. These tumors were therefore refractory to standard pathology protocols. Subsequent diagnosis was determined by analysis of whole-genome sequencing and RNA-Seq data, and validated by pathology review and immunohistochemistry.^7^ The prediction by SCOPE was compared against this putative diagnosis (eAppendix 3 in the Supplement). As shown in Figure 3, the classifier’s prediction matched all putative diagnoses except 1 Ewing sarcoma, 1 neuroendocrine tumor, and 1 salivary carcinoma; these 3 cancer types were not present in training.

## Discussion

We present a cancer-type classifier that leverages the entire gene-expression profile of a tumor sample. Our method achieves 97% overall accuracy and a mean (SD) *F*_1_ score of 0.92 (0.06) on our held-out set. This performance level is maintained on external cohorts, with an overall accuracy of 99% on primary cancers and mean (SD) accuracy of 86% (11%) for metastatic disease. We use the confidence score values (equivalent to probabilities) for predictions to characterize cancers with mixed histology (eFigure 9 in the Supplement).

Our findings support observations in literature that physiologically proximal and morphologically similar cancer types, such as stomach adenocarcinomas and some esophageal adenocarcinomas, are highly similar at the whole-transcriptome level, in spite of having distinct clinical designations.^15,34^ It also reflects the existing challenge with using glass-based pathology (even with the aid of immunohistochemistry) to discern these tumors.

We observed poorer performance of the method in pretreated metastases. This may be driven by known biological differences in the metastatic space. SCOPE also has difficulty discriminating metastatic cancers that share the same organ system of origin, if tumor content of the sequenced sample is low. It is possible that although there is diluted signal for the correct cancer type, low tumor content limits an accurate prediction. Incorporating more metastatic cancers in training should address these deficiencies.

### Limitations

A limitation of SCOPE is the lack of external validation sets for all classes. We intend to incorporate these data sets as they become available. A challenge for general application of this method is transcriptomic data that has been generated from RNA extracted from formalin-fixed paraffin-embedded (FFPE) tissue, rather than from snap frozen tissue. Formalin-fixed paraffin-embedded specimens are persistent morphologic records of tissue biopsies, and highly prevalent in pathology laboratories worldwide. However, controllable and uncontrollable variables, including tissue characteristics, fixation technique, and storage conditions, can affect the yield and quality of total RNA in FFPE blocks. We obtained 100% accuracy on 5 in-house primary FFPE samples. Nonetheless, FFPE application of this method will require additional validation.

Rare tumors are typically underrepresented in public data sets, which is currently a challenge in generalizing classification methods such as ours. They are also challenging to diagnose with conventional pathology methods. We show the utility of synthetic oversampling to legitimately generate additional training samples for rare cancers. Cancers of unknown primary site form 3% to 5% of all cancer diagnoses.^35^ This is a powerful method to identify diagnostic candidates where results from conventional pathology diagnosis are inconclusive.

## Conclusions

A challenging part of building molecular diagnostics is selection of relevant features. With recent advances in computational frameworks, we can manipulate high-dimensional data quickly and efficiently, allowing us to explore a machine learning approach that leverages large training sets across multiple tumor types. This is also a hypothesis-generating method for discovery of new diagnostic biomarkers. The method is available online.^36^

As demonstrated by previous studies, SCOPE has proven valuable for orthogonal assessment of common cancers^26^ and for contextualizing the biological features of complex, rare cancer types.^37^ As shown by its performance on CUPs, it is particularly useful in expediting precision oncology workflows and in clinical laboratories where access to a plethora of immunostains for sequential diagnosis may be limited.

## Notes

Supplement. **eAppendix 1.** Model Selection **eAppendix 2.** Training Data **eAppendix 3.** Test Data **eAppendix 4.** Metrics Used **eAppendix 5.** Data Pre-processing **eAppendix 6.** Feature Weight Analysis **eAppendix 7.** Differences in Performance on Held-out Set for Feature Selection Algorithms **eTable 1.** Cancer Types and Corresponding Abbreviations Used for Training, and Referenced in Text **eTable 2.** Breakdown of Cancer Types in the External Metastatic Cohort **eTable 3.** Architecture and Identifying Names for Each Neural Network That Makes up the SCOPE Ensemble Classifier **eTable 4.** Important Genes Based on Frequency Analysis of Gene Weights for Each Neural Network in SCOPE **eTable 5.** Detailed Breakdown of Prediction Trends in the Metastatic Cohort, With Classes of Mispredictions Listed **eTable 6.** Detailed Version of Table 2, Whereby the Performance of the Smaller Classes Has Been Described in Detail **eFigure 1.** Performance of Various Models (Distinct Based on Feature Selection and Architecture) on the Held-out Set **eFigure 2.** Performance of Algorithms on CV Folds During Training **eFigure 3.** Performance of SCOPE on the Held-out Set **eFigure 4.** The Performance of Individual Neural Networks on the Held-out Set **eFigure 5.** t-SNE Plot of Transcriptomic Data in TCGA Training Cohorts **eFigure 6.** t-SNE Plot of Transcriptomic Data in TCGA Training Cohorts **eFigure 7.** A Detailed Version of Figure 2A, Whereby the Smaller Classes Are Shown Individually Instead of in Aggregate **eFigure 8.** The Distribution of Values for Tests of Association Between Classification Accuracy and a) Tumor Content (%), b) Confidence Score, and c) Training Class Size Are Shown **eFigure 9.** Example Outputs From SCOPE **eReferences.**
