# Supplementary material for: Application of a Neural Network Whole Transcriptome–Based Pan-Cancer Method for Diagnosis of Primary and Metastatic Cancers
Source: JAMA Netw Open. 2019 Apr 26;2(4):e192597. doi: 10.1001/jamanetworkopen.2019.2597 (PMC6487574; doi:10.1001/jamanetworkopen.2019.2597)
Supplement: Supplement. — eAppendix 1. Model Selection eAppendix 2. Training Data eAppendix 3. Test Data eAppendix 4. Metrics Used eAppendix 5. Data Pre-processing eAppendix 6. Feature Weight Analysis eAppendix 7. Differences in Performance on Held-out Set for Feature Selection Algorithms eTable 1. Cancer Types and Corresponding Abbreviations Used for Training, and Referenced in Text eTable 2. Breakdown of Cancer Types in the External Metastatic Cohort eTable 3. Architecture and Identifying Names for Each Neural Network That Makes up the SCOPE Ensemble Classifier eTable 4. Important Genes Based on Frequency Analysis of Gene Weights for Each Neural Network in SCOPE eTable 5. Detailed Breakdown of Prediction Trends in the Metastatic Cohort, With Classes of Mispredictions Listed eTable 6. Detailed Version of Table 2, Whereby the Performance of the Smaller Classes Has Been Described in Detail eFigure 1. Performance of Various Models (Distinct Based on Feature Selection and Architecture) on the Held-out Set eFigure 2. Performance of Algorithms on CV Folds During Training eFigure 3. Performance of SCOPE on the Held-out Set eFigure 4. The Performance of Individual Neural Networks on the Held-out Set eFigure 5. t-SNE Plot of Transcriptomic Data in TCGA Training Cohorts eFigure 6. t-SNE Plot of Transcriptomic Data in TCGA Training Cohorts eFigure 7. A Detailed Version of Figure 2A, Whereby the Smaller Classes Are Shown Individually Instead of in Aggregate eFigure 8. The Distribution of Values for Tests of Association Between Classification Accuracy and a) Tumor Content (%), b) Confidence Score, and c) Training Class Size Are Shown eFigure 9. Example Outputs From SCOPE eReferences. [file jamanetwopen-2-e192597-s001.pdf]

Supplementary Online Content

Grewal JK, Tessier-Cloutier B, Jones M, et al. Application of a neural network whole transcriptome–based pan-cancer method for diagnosis of primary and metastatic cancers. *JAMA Netw Open*. 2019;2(4):e192597. doi:10.1001/jamanetworkopen.2019.2597

eAppendix 1. Model Selection
eAppendix 2. Training Data
eAppendix 3. Test Data
eAppendix 4. Metrics Used
eAppendix 5. Data Pre-processing
eAppendix 6. Feature Weight Analysis
eAppendix 7. Differences in Performance on Held-out Set for Feature Selection Algorithms
eTable 1. Cancer Types and Corresponding Abbreviations Used for Training, and Referenced in Text
eTable 2. Breakdown of Cancer Types in the External Metastatic Cohort
eTable 3. Architecture and Identifying Names for Each Neural Network That Makes up the SCOPE Ensemble Classifier
eTable 4. Important Genes Based on Frequency Analysis of Gene Weights for Each Neural Network in SCOPE
eTable 5. Detailed Breakdown of Prediction Trends in the Metastatic Cohort, With Classes of Mispredictions Listed
eTable 6. Detailed Version of Table 2, Whereby the Performance of the Smaller Classes Has Been Described in Detail
eFigure 1. Performance of Various Models (Distinct Based on Feature Selection and Architecture) on the Held-out Set
eFigure 2. Performance of Algorithms on CV Folds During Training
eFigure 3. Performance of SCOPE on the Held-out Set
eFigure 4. The Performance of Individual Neural Networks on the Held-out Set
eFigure 5. t-SNE Plot of Transcriptomic Data in TCGA Training Cohorts
eFigure 6. t-SNE Plot of Transcriptomic Data in TCGA Training Cohorts
eFigure 7. A Detailed Version of Figure 2A, Whereby the Smaller Classes Are Shown Individually Instead of in Aggregate
eFigure 8. The Distribution of Values for Tests of Association Between Classification Accuracy and a) Tumor Content (%), b) Confidence Score, and c) Training Class Size Are Shown
eFigure 9. Example Outputs From SCOPE
eReferences.

This supplementary material has been provided by the authors to give readers additional information about their work.

© 2019 Grewal JK et al. *JAMA Network Open*.

eAppendix 1. Model Selection

As the 66 training cohorts had a wide range of representative samples (3-1020), using accuracy as a metric of performance of the classifiers would not necessarily demonstrate the ability of the classifier to discriminate between all 66 output classes. Therefore, the performance of the trained models was evaluated using the F1-score as a measure. The F1-score is the harmonic mean of precision and recall, with 1.0 being the most desirable value (precision and recall of 1.0 for all output categories), and 0 representing a classifier that has 0 precision and 0 recall on all output categories.

Algorithmic Model Selection

Several supervised learning algorithms were evaluated, including a baseline linear comparator discussed below. For the selection of an initial classifier based on the training data, we evaluated the performance of 4 supervised learning methods, namely multi-class support vector machines (SVM), random forests (RF), extra trees ensemble (ET), and an ensemble of neural networks. Figure 1 shows the cross-validation results of the best performing model in each classification algorithm category. The optimal models for these comparators were selected with grid search across a space of model-specific parameters, using 5-CV to identify the best model. Shallow (1 hidden layer) feed-forward neural networks were trained with varying sizes of the hidden layer. In the selection of the optimal parameter set for the neural networks, our search space spanned a range of values for the learning rate and the regularizers (L1 and L2).

SVMs, RFs, and ETs were implemented using the scikit-learn package in python, and training was done on CPU machines. The custom neural nets were defined and trained using the lasagne library[6] in Python, and training was done using NVIDIA GEForce GTX TITAN X GPUs. The F1-Score was used as the main metric of assessment, to account for class imbalances in the training and test sets.

Baseline Linear Comparator

Using pairwise ANOVA, 3000 genes were found to be significantly enriched for distinguishing between the 66 training tissue and cancer types. Cancer samples in the metastatic cohort were evaluated by calculating simple pair-wise spearman correlation for these genes, for every test sample versus the TCGA samples (separated by cancer type). The highest correlated cancer/normal type was chosen as the predicted class by this approach.

SCOPE Ensemble Construction

Firstly, we found that synthetic minority oversampling (SMOTE) [5] resulted in improved classification of rare classes as compared to training with the class imbalanced dataset (eFigure 3).

© 2019 Grewal JK et al. *JAMA Network Open*.

Secondly, comparison of different data normalizations showed that using rank transformed RPKM-improved classification accuracy for the overall model. Based on these results, we extended the classifier into an ensemble of neural networks combining these data transformation techniques. The additional neural networks were selected using 5-CV. We also observed that each of these 5 machines was better at classifying different classes within the training dataset, reflecting that they have each learnt different (unknown) modalities for classification of the 66 different tissue and cancer types. Based on this observation, we adopted weighted majority voting approach was used to obtain predictions for new samples from the ensemble. This resulted in a re-weighted vector of 66 values from each network. These reweighted predictions were then pooled using a majority-voting approach, providing an averaged probability score and a similarity confidence for each class.

The architecture and details of data pre-processing of each network are as described in eTable 3. Learning rate was 0.001, with early stopping when validation cost increased for more than 3 epochs, or was stable for 3 continuous epochs. Maximum number of allowed epochs was 1000, thus training went until 1000 epochs or early stopping threshold, whichever came first.

© 2019 Grewal JK et al. *JAMA Network Open*.

eAppendix 2. Training Data

Multi-platform RNA-Seq data was obtained from The Cancer Genome Atlas (TCGA, multi-platform - Illumina Hi-Seq 2000 and Genome Analyzer II, processed with TCGA RNA-Seq v2 RSEM processing pipeline), the National Cancer Institute (NCI) non-Hodgkin lymphoma dataset [1] (sequenced with Illumina Genome Analyzer II, median normalized), and non- cell-line primary tumor data from the Terry Fox Research Institutes Glioblastoma Multiforme (TFRI-GBM) project. 2 in-house cancer cohorts, adult medulloblastoma (MB-Adult) and follicular lymphoma (FL), further supplemented this dataset (sequenced with Illumina HiSeq 2500). Colon and rectum adenocarcinomas from TCGA were combined into a single cohort (COADREAD) due to their geographical proximity in primary lesions, supported by findings from our initial quality control that showed insufficient decomposition of these two cancer types based on their transcriptomic data. The TCGA RNA-Seq libraries were prepared by various different sequencing centers, but to facilitate harmonization across samples, the TCGA RNA-Seq v2 RSEM processing pipeline treated all RNA-Seq reads as unstranded. This resulted in a dataset of 10,822 transcriptomes spanning 40 different untreated primary tumor types and 26 adjacent normal tissue types (66 classes), with individual class sizes ranging from 3 to 1095 samples (**eTable** 1). No feature selection was done on the consolidated set of transcriptomes besides filtering for (a) genes with a recorded RPKM value in every sample (n=21,220), and (b) genes that overlapped with available annotations for our independent test sets (n=17,688). This resulted in a set of 10,822 samples, spanning 66 different tumor and adjacent normal classes, and with each sample represented by 17,688 distinct median normalized gene RPKM values. **eTable** 1 shows the annotations used, following TCGA nomenclature. The table also shows the number of training samples available for each cancer type.

Training data was randomly split up into 4/5th model training data, and 1/5th held-out test data. The training and test splits maintained relative class frequencies. In classes with less than 5 samples (6 classes, all adjacent normal), 1 sample was randomly assigned to the held-out test set, and the remaining samples were kept in the model training set. All models discussed in this paper were trained on the 4/5th model training data, with the held-out test set used as the first external validation of performance of the fully trained models. Stratified 5-fold Cross Validation (5-CV) was used for hyperparameter selection for each algorithm. The StratifiedKFold function in the scikit-learn package in Python was used to generate class-balanced CV folds [2].

© 2019 Grewal JK et al. *JAMA Network Open*.

eAppendix 3. Test Data

Primary Mesotheliomas

Mesothelioma is a rare and aggressive cancer arising in the linings of lung, abdomen, or the heart. The Genentech Mesothelioma dataset has 211 transcriptomes from untreated, primary lung biopsies of mesothelioma, spanning 4 distinct molecular subtypes of mesothelioma sarcomatoid (n=29), epitheliod (n=54), biphasic-epitheliod (n=72), and biphasic-sarcomatoid (n=56). The RNA-seq libraries were pre- pared using TruSeq RNA Sample Preparation kit (unstranded, polyA+) from Illumina, and sequenced on the HiSeq 2500 (66 million paired-end reads per sample) [3].

Metastatic disease and CUPs

The Personalized OncoGenomics (POG) project at the BC Cancer Agency (BCCA) was established in 2011 with the aim to sequence patients with cancers that no longer respond to standard treatment [4]. The project analyses the genomic and gene expression (transcriptomic) data of each patient, in order to identify drugs that can target the individual cancer. The vast majority of patients enrolled into POG presented with metastatic disease with a known primary diagnosis and location. Biopsies are collected from the metastatic site in most cases. In a minority of cases, the tissue is taken from the original site of disease presentation (noted as such in main text, Table 2). This could be due to the intrinsic biology of the cancer in the form of LGG and GBM, or from multiple factors including site and size of lesion. Clinical laboratories at the BCCA assess the site of origin of POG cancer biopsies according to established protocols. The primary role of the pathologist in this context is to perform quality assurance of the tissue, in the determination of cellularity and percentage tumor content, to assist in downstream genomic interpretation. Secondarily, she reviews the patient’s clinical history for information pertaining to other archival pathology specimens particularly the initial untreated diagnostic tissue, often obtained from the primary site. Histological information of that tissue should match the subsequent specimen from the metastatic site. Typically, protein expression pattern, in the form of immunohistochemical (IHC) profile, should be similar but not necessarily identical. Lineage- specific markers that define the cell- of- origin of the tumor, such as specific cytokeratin markers, should be stable in its expression regardless of site. However, other biomarkers are more dynamic or labile, and their changes could represent alteration in the state of differentiation (for example, neuroendocrine dedifferentiation, malignant transformation) of the tumor or changes in the hormonal status and both could have significant impact on the biology of the

© 2019 Grewal JK et al. *JAMA Network Open*.

tumor and its management. A minimum pathology estimated tumor content of 40% in the tissue section is required prior to sequencing. Subsequently, standard Illumina protocols are followed for whole genome sequencing (WGS) for the tumor and peripheral blood (as control), and transcriptome sequencing for the tumor. The RNA-Seq libraries were prepared using strand specific RNA-Seq (ssRNA-Seq) Sample Preparation kit (stranded, polyA+) from Illumina, and sequenced on the Illumina HiSeq 2500 (200 million paired-end reads per sample). For the analysis presented in this paper, metastatic cases were selected from the POG cohort based on the following criteria (a) a primary of origin was identified, based on a joint consideration of clinical/pathology/genomic data, and (b) cDNA libraries prepared from the biopsy sample passed in-house quality control. Based on these criteria, we identified 201 samples spanning 26 different cancer types, summarized in **(eTable 2)**. eTable 2 shows the annotations used, following TCGA nomenclature. The table also shows the number of training samples available for each cancer type.

Additionally, the POG cohort contained 16 cases where the primary site of origin could not be determined by initial pathology analysis. Genomic and transcriptomic analysis as part of the POG project determined the corresponding cancer type for 15 of these cases, which was used as gold standard for assessing the prediction from the classifier. The classification was performed retroactively after the closest suitable cancer type had been determined based on detailed pathway-level and genomic analyses of the cancer.

© 2019 Grewal JK et al. *JAMA Network Open*.

eAppendix 4. Metrics Used

Since the trained cancer and normal cohorts had variable representation in the training set (3 for adjacent normal subcutaneous melanoma, to 1095 for breast cancer), we used the F1-score as a measure of overall performance of the classifier. The F1-score is the harmonic mean of precision and recall, with 1.0 being the most desirable value (precision and recall of 1.0 for all output categories), and 0 representing a classifier that has 0 precision and 0 recall on all output categories.

For a given input, the ensemble generates a pooled confidence score for each of the 66 output classes. Predicted classes are jointly ordered by the confidence score and number of machines in agreement. This max vote-pooling method was used to obtain a quantitative confidence score for each category. This confidence score was taken as a proxy for differential diagnosis when assessing metastatic samples. Thus, in the event that the prediction from the ensemble classifier was split between different cancer types, the correctness of the prediction was assessed by comparing the diagnosed cancer type against the pool of confident predictions.

© 2019 Grewal JK et al. *JAMA Network Open*.

eAppendix 5. Data Pre-processing

Data normalization

Technical artifacts in the training data can be amplified if not filtered out, resulting in over-fitting while training a classifier. This results in a classifier that performs quite well on the training data, but does not generalize to samples that it has not seen during training. Data transformation is one way to avoid over fitting to the input data. This is generally done by re-scaling the input data to fall within a certain range of values, or by forcing it to follow a certain distribution (ex. normal distribution for expression data). We assessed the utility of data transformation in improving the best performing model, the shallow neural network trained with RPKM input. To this end, various scaling and data transformation methods, namely minmax scaling, L2 norm scaling, and rank normalization (average), were assessed separately. The performance of each approach was assessed by stratified 5-CV. Our assessment of normalization methods and feature selection (vs using the entire transcriptome) showed that (a) the actual RPKM profile across the 17,688 genes has a better classification performance than using feature selection or feature subsetting to known cancer-associated genes, and that (b) there is a performance gain with rank normalization of RPKM data prior to training.

Class expansion

A supervised machine learning based classifier works by seeing multiple different samples representing each cancer/tissue type and steadily learning which genes (features) are most valuable in identifying each type of interest. A common problem with this approach is that a classifier can sometimes fail to appreciate the features that characterize the smaller cancer/tissue types. This class imbalance can be overcome by pre-processing the training set in specific ways by duplicating some of the samples in the smaller class(es), by punishing the classifier more for making a mistake with the smaller classes, or by supplementing the smaller classes with synthetic samples. One such method for adding synthetic samples to smaller classes is Synthetic Minority Oversampling (SMOTE). We trained and assessed the performance of the RPKM-based neural network classifier method using 3 different class expansion approaches, (a) duplicating samples randomly in the smaller cohorts to inflate their total sample size to the largest class, (b) adding an inverse weight factor for mis-classification of smaller classes (i.e. making it more expensive for the classifier to mislabel a sample from a smaller class during training), (c) adding synthetic samples using SMOTE, and compared these 3 approaches to (d) doing no class expansion. Duplicated/synthetic samples were only added to the training folds,

© 2019 Grewal JK et al. *JAMA Network Open*.

so that the cross-validation test fold always only contained non-synthetic samples that were absent in the training folds. The synthetic sampling algorithm is as adapted from Chawla et al [5]. The results over stratified 5-CV for each type of class expansion showed that there was an increase in overall F-score when SMOTE was used to expand the training folds.

© 2019 Grewal JK et al. *JAMA Network Open*.

eAppendix 6. Feature Weight Analysis

Following training of each neural network, the weights and biases for the fully connected layers were extracted using the lasagne.layers.get˙all˙param˙values(network) function. Subsequently, following the rules of weight propagation in fully connected neural networks, a forward multiplication loop was evaluated, resulting in a matrix of dimensions [Number of genes, Number of output categories]. For each output category, the resultant network weights were sorted, and the top-100 genes with the highest weights for the class were saved. This was done over 5-cross validation models for each neural network, resulting in 25 lists of top-100 genes. For a given neural network, genes found to be top-ranked in atleast 3 out of 5 CV folds were identified. Subsequently, for each category, the NN-specific top genes were filtered for occurrence in atleast 3/5 neural networks, resulting in the presented list of important genes in each class (eTable 4).

© 2019 Grewal JK et al. *JAMA Network Open*.

eAppendix 7. Differences in Performance on Held-out Set for Feature Selection Algorithms

As can be seen in main Figure 1(A), the performance of the different models is better on the held-out set than that quantified through cross-validation. This is because cross-validation only happens on 80% of the data, which in turn is split into 80% training and 20% cross-validation test fold. This impacts the smaller classes, which have fewer samples to train on in a cross-validation run. As a result, the performance of the classifier is poorer on the cross-validation test folds for such classes. However, when testing on the 20% heldout set, we are training the model on the entire 80% of the data. While this is of little consequence to classes that are well represented, the smaller classes are more thoroughly learnt during the training. This impact is evident in eFigure 6.

© 2019 Grewal JK et al. *JAMA Network Open*.

eTable 1. Cancer Types and Corresponding Abbreviations Used for Training, and Referenced in Text.

The training cohort sizes are indicated for the adjacent normal and primary tumor.

**Abbreviation**	**Full Name**	**Case count – Normal**	**Case count - Tumor**
ACC	Adrenocortical Carcinoma	-	79
BLCA	Urothelial Bladder Carcinoma	19	408
BRCA	Breast Ductal Carcinoma	113	1095
CESC_CAD	Cervical and Endocervical Adenocarcinoma	3	47
CESC_SCC	Cervical Squamous Cell Carcinoma	6	257
CHOL	Cholangiocarcinoma	27	36
COADREAD	Colorectal Adenocarcinoma	51	372
DLBC	Diffuse Large B-Cell Lymphoma	-	48
DLBC_BM	DLBCL Blood/Bone Marrow	-	11
ESCA	Esophageal Carcinoma	3	15
ESCA_EAC	Esophageal Adenocarcinoma	24	79
ESCA_SCC	Esophageal Squamous Cell Carcinoma	6	90
FL	Follicular Lymphoma	-	50
GBM	Glioblastoma Multiforme	15	161
HNSC	Head and Neck Squamous Cell Carcinoma	44	520
KICH	Chromophobe Renal Cell Carcinoma / Kidney Chromophobe	25	66
KIRC	Renal Clear Cell Carcinoma	72	533
KIRP	Papillary Renal Cell Carcinoma	32	290
LAML	Acute Myeloid Leukemia	-	173
LGG	Lower Grade Glioma	-	516
LIHC	Liver Hepatocellular Carcinoma	50	371
LUAD	Lung Adenocarcinoma	59	515
LUSC	Lung Squamous Cell Carcinoma	50	501
MB-Adult	Adult Medulloblastoma	-	143
MESO	Mesothelioma	-	87
NCI_GPH_DLBCL	Diffuse Large B-Cell Lymphoma (NCI cohort)	-	111
OV	Ovarian Serous Cystadenocarcinoma	-	305
PAAD	Pancreatic Ductal Adenocarcinoma	12	178
PCPG	Paraganglioma & Pheochromocytoma	9	179
PRAD	Prostate Adenocarcinoma	52	497
SARC	Sarcoma	6	259
SKCM	Cutaneous Melanoma	3	469
STAD	Stomach Adenocarcinoma	35	415
TFRI_GBM_NCL	Glioblastoma Multiforme (TFRI cohort)	-	52
TGCT	Testicular Germ Cell Cancer	-	150
THCA	Thyroid Carcinoma	59	505
THYM	Thymoma	6	120
UCEC	Uterine Corpus Endometrial Carcinoma	24	177
UCS	Uterine Carcinoma	-	57
UVM	Uveal Melanoma	-	80
		**805**	**10,017**

© 2019 Grewal JK et al. *JAMA Network Open*.

eTable 2. Breakdown of Cancer Types in the External Metastatic Cohort

**Abbreviation**	**Organ System**	**Full Name**	**Case count**
ACC	Endocrine	Adrenocortical Carcinoma	2
BRCA	Breast	Breast Ductal Carcinoma	69
CESC_CAD	Gynecologic	Cervical and Endocervical Adenocarcinoma	1
CHOL	Gastrointestinal	Cholangiocarcinoma	5
COADREAD	Gastrointestinal	Colorectal Adenocarcinoma	22
DLBC	Hematologic	Diffuse Large B-Cell Lymphoma	1
ESCA_EAC	Gastrointestinal	Esophageal Adenocarcinoma	2
ESCA_SCC	Gastrointestinal	Esophageal Squamous Cell Carcinoma	4
FL	Hematologic	Follicular Lymphoma	1
GBM	Central Nervous System	Glioblastoma Multiforme	4
KIRP	Urologic	Papillary Renal Cell Carcinoma	2
LIHC	Gastrointestinal	Liver Hepatocellular Carcinoma	1
LGG	Central Nervous System	Lower Grade Glioma	2
LUAD	Thoracic	Lung Adenocarcinoma	18
LUSC	Thoracic	Lung Squamous Cell Carcinoma	1
MESO	Thoracic	Mesothelioma	5
OV	Gynecologic	Ovarian Serous Cystadenocarcinoma	7
PAAD	Gastrointestinal	Pancreatic Ductal Adenocarcinoma	11
PRAD	Urologic	Prostate Adenocarcinoma	1
SARC	Soft Tissue	Sarcoma	23
SKCM	Skin	Cutaneous Melanoma	3
STAD	Gastrointestinal	Stomach Adenocarcinoma	3
TGCT	Urologic	Testicular Germ Cell Cancer	1
THYM	Hematologic	Thymoma	1
UCEC	Gynecologic	Uterine Corpus Endometrial Carcinoma	6
UCS	Gynecologic	Uterine Carcinoma	5
			**201**

© 2019 Grewal JK et al. *JAMA Network Open*.

eTable 3. Architecture and Identifying Names for Each Neural Network That Makes up the SCOPE Ensemble Classifier.

**Model Name**	**Architecture**	**Data pre-processing**	**Additional Rules**
None17k	17688 x 17000 x 66	None (RPKM)	None
None17kDropout	17688 x 17000 x 17000 x 66	None (RPKM)	Dropout (10%) input in training
SmoteNone17k	17688 x 17000 x 66	None (RPKM) + SMOTE samples	None
Rm500	17688 x 500 x 66	Rank norm + Minmax(0,1) scaling	None
Rm500Dropout	17688 x 500 x 500 x 66	Rank norm + Minmax(0,1) scaling	Dropout (10%) input in training

© 2019 Grewal JK et al. *JAMA Network Open*.

eTable 4. Important Genes Based on Frequency Analysis of Gene Weights for Each Neural Network in SCOPE

Abbreviation	Malignancy	Organ System	Important Genes
ACC	Tumor	Endocrine	CYP11A1, CYP17A1, CYP21A2, DLK1, GSTA1, IGF2, NPTX2, STAR
BLCA	Adjacent Normal	Urologic	ACTG2, CNN1, DES, DHRS2, GPX2, KRT13, KRT5, LY6D, OLFM4, PLA2G2A, S100P, SPRR3, UPK2
BLCA	Tumor	Urologic	AKR1C2, DES, DHRS2, GATA3, GPX2, KRT13, KRT17, KRT5, PSCA, S100P, SPINK1, UPK1B, UPK2
BRCA	Adjacent Normal	Breast	ADH1B, ADIPOQ, APOD, AZGP1, GATA3, KRT14, LPL, MUCL1, PIP, PLIN1, S100A1, SAA1, SCGB1D2, SCGB2A2, TFF1
BRCA	Tumor	Breast	AGR3, AZGP1, CALML5, CRABP2, EFHD1, FABP4, GATA3, KRT14, KRT6B, LTF, MMP11, MUCL1, NPY1R, PIP, SCGB2A2, SERPINA3, SPDEF, TFF1
CESC_CAD	Adjacent Normal	Gynecologic	DES
CESC_CAD	Tumor	Gynecologic	CEACAM5, CLDN3, KRT7, MMP11, PIGR, SCGB2A1
CESC_SCC	Adjacent Normal	Gynecologic	CNN1
CESC_SCC	Tumor	Gynecologic	CALML3, KRT13, KRT14, KRT19, KRT5, KRT6A, MMP11
CHOL	Tumor	Gastrointestinal	AGT, ALB, AMBP, CEACAM6, CRP, FGA, FGB, FGG, ORM1, REG1A, TM4SF4, TTR
COADREAD		Gastrointestinal	AQP8, CA1, CEACAM7, CLCA1, DES, FABP1, FAM3D, GPX2, GUCA2A, KRT20, SLC26A3, SPINK4, ZG16
COADREAD		Gastrointestinal	CDH17, CDX2, CEACAM5, CEACAM6, DPEP1, FABP1, FAM3D, GPX2, LGALS4, MUC13, MUC2, PLA2G2A, PPP1R1B, REG4, S100P, SPINK4, TSPAN8, VIL1
DLBC	Tumor	Hematologic	-
DLBC_BM	Tumor	Hematologic	-
ESCA	Adjacent Normal	Gastrointestinal	KRT13
ESCA	Tumor	Gastrointestinal	CLDN18, CST1, MALAT1, REG1A, REG3A, SPINK1
ESCA_EAC	Adjacent Normal	Gastrointestinal	ACTG2, DES, LIPF, PGA3, PGA4
ESCA_EAC	Tumor	Gastrointestinal	CEACAM5, CST1, KRT13, LGALS4, MALAT1, MUC13, PIGR, PLA2G2A, S100A7, SPRR3, TSPAN8, UBD
ESCA_SCC	Adjacent Normal	Gastrointestinal	SPRR1B
ESCA_SCC	Tumor	Gastrointestinal	CALML3, CST1, DES, KRT14, KRT5, LY6D, MALAT1, S100A7, SPRR1B, SPRR3, TRIM29
FL	Tumor	Hematologic	CCL21
GBM	Tumor	Central Nervous System	AQP4, CHI3L1, GFAP
HNSC	Adjacent Normal	Head and Neck	ACTA1, CALML5, CKM, KRT13, KRT4, MB, MUC7, MYH2, MYL1, MYL2, MYLPF, PIP, PRB3, SAA1, SCGB3A1, SMR3B, STATH, TCAP, TGM3, TNNC2

© 2019 Grewal JK et al. *JAMA Network Open.*

HNSC	Tumor	Head and Neck	ACTA1, CALML3, CALML5, KRT13, KRT14, LGALS7, MMP1, SPRR2A, SPRR3
KICH	Adjacent Normal	Urologic	ALDOB, AQP2, FXYD2, UMOD
KICH	Tumor	Urologic	ATP6V0A4, ATP6V0D2, CDH16, DEFB1, RHCG, SPINK1, SPP1, TMEM213
KIRC	Adjacent Normal	Urologic	AQP2, CDH16, SLC34A2, UMOD
KIRC	Tumor	Urologic	ANGPTL4, CA12, CA9, DEFB1, EGLN3, ESM1, FXYD2, GSTA1, NAT8
KIRP	Adjacent Normal	Urologic	AQP2, PIGR, UMOD
KIRP	Tumor	Urologic	C19orf33, MAL, MMP7, PIGR, SST, WFDC2
LAML	Tumor	Hematologic	AZU1, CSF3R, FOSB, MPO, PRTN3, RNASE2, S100A8
LGG	Tumor	Central Nervous System	EEF1A1P9, GFAP, PTPRZ1
LIHC	Adjacent Normal	Gastrointestinal	IGFBP1
LIHC	Tumor	Gastrointestinal	ALB, APCS, APOA2, APOC3, CRP, FGA, FGB, GC, HULC, ITIH2, RBP4, TF, TM4SF4, UBD, VTN
LUAD	Adjacent Normal	Thoracic	HBA1, NAPSA, SCGB1A1, SCGB3A1, SCGB3A2, SFTPA1, SFTPB, SFTPC, SFTPD, SLPI
LUAD	Tumor	Thoracic	C8orf4, CEACAM5, CRABP2, FGG, NAPSA, PGC, S100P, SCGB1A1, SCGB3A1, SCGB3A2, SFTA2, SFTPA1, SFTPA2, SFTPB, SLC34A2, SPINK1
LUSC	Adjacent Normal	Thoracic	CCL21, NAPSA, RPS4Y1, SCGB1A1, SCGB3A1, SCGB3A2, SFTA2, SFTPA1, SFTPA2, SFTPB, SFTPC, SFTPD, SLC34A2
LUSC	Tumor	Thoracic	AKR1C2, CALML3, CES1, KRT15, KRT16, KRT19, KRT5, KRT6A, KRT6B, NAPSA, NTS, SCGB1A1, SCGB3A2, SFTPA1, SFTPA2, SFTPB, SFTPC, SPRR2A
MB-Adult	Tumor	Central Nervous System	GFAP, STMN2
MESO	Tumor	Thoracic	C19orf33, CALB2, EFEMP1, ITLN1, KRT19, KRT7, MSLN, UPK3B
NCI_GPH_DLBCL	Tumor	Hematologic	-
OV	Tumor	Gynecologic	CHI3L1, CLDN3, FOLR1, KLK6, KLK7, MALAT1, MSLN, PAX8, SCGB2A1, SOX17, SST
PAAD	Adjacent Normal	Gastrointestinal	CELA3A, CPA1, CPB1, CRP, CTRB1, CTRB2, CTRC, CTSE, GCG, INS, PNLIP, PPY, PRSS1, REG1A, REG3A, TTR
PAAD	Tumor	Gastrointestinal	AGR2, CEACAM5, CHGB, CTRB1, CTRB2, GCG, INS, PNLIP, PPY, REG1A, REG4, S100P, SFRP2, SPINK1, SST, TFF1, TFF2, TTR
PCPG	Adjacent Normal	Endocrine	CYP11B1, CYP17A1, DLK1, GSTA1, STAR
PCPG	Tumor	Endocrine	CHGA, CHGB, DBH, DLK1, NPY, PENK
PRAD	Adjacent Normal	Urologic	ACPP, KLK2, KLK3, KLK4, NPY, OLFM4, PIP, SEMG1

© 2019 Grewal JK et al. *JAMA Network Open*.

PRAD	Tumor	Urologic	ACTG2, AZGP1, DES, FOLH1, FOXA1, KLK2, KLK3, KLK4, NKX3-1, NPY, PLA2G2A, SLC45A3
SARC	Tumor	Soft Tissue	DLK1
SKCM	Adjacent Normal	Skin	DCT, MLANA, PRAME, TYR
SKCM	Tumor	Skin	APOD, DCT, EDNRB, KRT6B, MLANA, PLP1, PRAME, S100A1, SERPINE2, SOX10, TYR, TYRP1, VGF
STAD	Adjacent Normal	Gastrointestinal	ACTG2, APOA1, APOA4, CLDN18, DES, GKN1, HSPB6, PGA4, PGC, PI3, REG3A
STAD	Tumor	Gastrointestinal	ACTG2, CEACAM6, CST1, MALAT1, PGC, REG4, SPINK1, TFF1, TFF3
TFRI_GBM_NCL	Tumor	Central Nervous System	MALAT1, PCDHGA1, PCDHGA8, PCDHGC4, PMP2
TGCT	Tumor	Urologic	DPPA3, DPPA5, GDF3, NANOG, POU5F1
THCA	Adjacent Normal	Endocrine	CCL21, HBA2, MT1G, PAX8, TG, TPO
THCA	Tumor	Endocrine	C16orf89, CLIC3, NKX2-1, S100A1, SFTA3, SFTPB, TG, TPO, ZCCHC12
THYM	Adjacent Normal	Hematologic	CALML3, CCL25, KRT5
THYM	Tumor	Hematologic	CALML3, CCL25, DNTT, KRT14, KRT15, KRT17, KRT19, KRT5, PAX1
UCEC	Adjacent Normal	Gynecologic	CNN1, DES
UCEC	Tumor	Gynecologic	MMP11, MSX1, PAX8, SCGB1D2, SCGB2A1, SFN, VTCN1
UCS	Tumor	Gynecologic	CRABP1, DLK1, PCOLCE, PRAME
UVM	Tumor	Heand and Neck	CITED1, MLANA, SOX10, TYR, TYRP1

© 2019 Grewal JK et al. *JAMA Network Open*.

eTable 5. Detailed Breakdown of Pprediction Trends in the Metastatic Cohort, With Classes of Mispredictions Listed**.**
Number of cases mispredicted as a specific classes are listed alongside in brackets.
[a]Precision, as indicated, is equivalent to class-specific accuracy.
[b]Prediction categories: Cases where predicted cancer type matched pathology diagnosis (Diagnosis) / was same as tissue type of biopsy site (Biopsy Site) / matched a cancer type with same organ-system of origin (Organ-system) / did not match any of the above (Other).
AC = "Adenocarcinoma", CA = "Carcinoma", SCC = "Squamous Cell Carcinoma", CESC – AC = "Cervical/Endocervical Adenocarcinoma", UCEC= "Uterine Corpus Endometrial Carcinoma"
GEJ_group: Esophageal AC, Esophageal SCC, Stomach AC, Liver Hepatocarcinoma, Papillary Kidney CA

		**Cohort metrics[a]**			**Count of cases predicted as[b]**			
**Diagnosed Type**	**Total Cases**	**Precision**	**Recall**	**F1-Score**	**Diagnosis**	**Biopsy Site**	**Organ-system**	**Other**
**Metastatic Site Biopsies**								
Adenocortical CA	1	1.00	1.00	1.00	1	-	-	-
Follicular Lymphoma	1	1.00	1.00	1.00	1	-	-	-
Mesothelioma	1	1.00	1.00	1.00	1	-	-	-
Prostate AC	1	1.00	1.00	1.00	1	-	-	-
Testicular Germ Cell Tumor	1	1.00	1.00	1.00	1	-	-	-
Thymoma	1	1.00	1.00	1.00	1	-	-	-
Colorectal AC	21	1.00	0.81	0.89	17	LIHC	STAD(2)	CHOL_n
Papillary Kidney AC	2	1.00	0.50	0.67	1	-	-	LUAD
UCEC	5	1.00	0.40	0.57	2	-	BRCA	BLCA,STAD
Uterine Carcinosarcoma	4	1.00	0.25	0.40	1	-	OV, SARC	HNSC
Breast CA	65	0.97	0.97	0.97	63	LIHC_n	-	BLCA
Lung AC	14	0.93	1.00	0.97	14	-	-	-
Sarcoma	17	0.90	0.53	0.67	9	LIHC	-	BRCA, DLBC (2), GBM, SKCM (2), KIRC
Ovarian CA	7	0.86	0.86	0.86	6	-	-	PAAD
Prostate AC	9	0.75	0.33	0.46	3	LIHC	CHOL (3), LUSC	BLCA
Cholangio-CA	5	0.67	0.80	0.73	4	-	STAD	-
Cutanous Melanoma	2	0.50	1.00	0.67	2	-	-	-
Diffuse Large B-Cell Lymphoma	1	0.33	1.00	0.50	1	-	-	-
Stomach AC	3	0.25	0.67	0.36	2	LIHC	-	-
CESC-AC	1	0.00	0.00	0.00	-	-	-	STAD
Esophageal AC	2	0.00	0.00	0.00	-	LIHC	STAD	-
Esophageal SCC	4	0.00	0.00	0.00	-	LUSC(1)	-	CESC_SCC (2), LUSC (1)
**Primary Site Biopsies**								
Adrenocortical CA	1	1.00	1.00	1.00	1	-	-	-

© 2019 Grewal JK et al. *JAMA Network Open*.

Breast CA	4	1.00	1.00	1.00	4	-	-	-
Colorectal AC	1	1.00	1.00	1.00	1	-	-	-
Glioblastoma Multiforme	4	1.00	1.00	1.00	4	-	-	-
Brain Glioma	2	1.00	1.00	1.00	2	-	-	-
Liver Hepatocarcinoma	1	1.00	1.00	1.00	1	-	-	-
Pancreatic AC	2	1.00	1.00	1.00	2	-	-	-
Cutaneous Melanoma	1	1.00	1.00	1.00	1	-	-	-
Uterine Carcinosarcoma	1	1.00	1.00	1.00	1	-	-	-
Sarcoma	6	1.00	0.83	0.91	5	-	-	HNSC
Lung AC	4	1.00	0.75	0.86	3	-	LUSC	-
Mesothelioma	4	1.00	0.75	0.86	3	-	-	KIRC
Lung SCC	1	0.50	1.00	0.67	1	-	-	-
UCEC	1	0.00	0.00	0.00	-	-	CESC_SCC	-
	**201**	**0.80**	**0.76**	**0.75**	**160**	**7**	**13**	**21**

© 2019 Grewal JK et al. *JAMA Network Open*.

eTable 6. Detailed Version of Table 2, Whereby the Performance of the Smaller Classes Has Been Described in Detail**.**
[a]TP = “True Positive Count”, TN = “True Negative Count”, FP = “False Positive Count”, FN = “False Negative Count”
Precision, as indicated, is equivalent to class-specific accuracy.
[b]Prediction categories: Cases where predicted cancer type matched pathology diagnosis (Diagnosis) / was same as tissue type of biopsy site (Biopsy Site) / matched a cancer type with same organ-system of origin (Organ-system) / did not match any of the above (Other)

AC = “Adenocarcinoma”, CA = “Carcinoma”, SCC = “Squamous Cell Carcinoma”, CESC – AC = “Cervical/Endocervical Adenocarcinoma”, UCEC= “Uterine Corpus Endometrial Carcinoma”
GEJ_group: Esophageal AC, Esophageal SCC, Stomach AC, Liver Hepatocarcinoma, Papillary Kidney CA

		**Cohort metrics[a]**							**Count of cases predicted as[b]**			
**Diagnosed Type**	**Total Cases**	**TP**	**TN**	**FP**	**FN**	**Precision**	**Recall**	**F1-Score**	**Diagnosis**	**Biopsy Site**	**Organ-system**	**Other**
**Metastatic Site Biopsies**												
Adenocortical CA	1	1	130	0	0	1.00	1.00	1.00	1	-	-	-
Follicular Lymphoma	1	1	130	0	0	1.00	1.00	1.00	1	-	-	-
Mesothelioma	1	1	130	0	0	1.00	1.00	1.00	1	-	-	-
Prostate AC	1	1	130	0	0	1.00	1.00	1.00	1	-	-	-
Testicular Germ Cell Tumor	1	1	130	0	0	1.00	1.00	1.00	1	-	-	-
Thymoma	1	1	130	0	0	1.00	1.00	1.00	1	-	-	-
Colorectal AC	21	17	114	0	4	1.00	0.81	0.89	17	1	2	1
Papillary Kidney AC	2	1	130	0	1	1.00	0.50	0.67	1	-	-	1
UCEC	5	2	129	0	3	1.00	0.40	0.57	2	-	1	2
Uterine Carcinosarcoma	4	1	130	0	3	1.00	0.25	0.40	1	-	2	1
Breast CA	65	63	68	2	2	0.97	0.97	0.97	63	1	-	1
Lung AC	14	14	117	1	0	0.93	1.00	0.97	14	-	-	-
Sarcoma	17	9	122	1	8	0.90	0.53	0.67	9	1	-	7
Ovarian CA	7	6	125	1	1	0.86	0.86	0.86	6	-	-	1
Pancreatic AC	9	3	128	1	6	0.75	0.33	0.46	3	1	4	1
Cholangio-CA	5	4	127	2	1	0.67	0.80	0.73	4	-	1	-
Cutanous Melanoma	2	2	129	2	0	0.50	1.00	0.67	2	-	-	-
Diffuse Large B-Cell Ly.	1	1	130	2	0	0.33	1.00	0.50	1	-	-	-
Stomach AC	3	2	129	6	1	0.25	0.67	0.36	2	1	-	-
CESC-AC	1	0	131	0	1	0.00	0.00	0.00	-	-	-	1
Esophageal AC	2	0	131	0	2	0.00	0.00	0.00	-	1	1	-
Esophageal SCC	4	0	131	0	4	0.00	0.00	0.00	-	1	0	3
**Primary Site Biopsies**												
Adrenocortical CA	1	1	28	0	0	1.00	1.00	1.00	1	-	-	-
Breast CA	4	4	25	0	0	1.00	1.00	1.00	4	-	-	-
Colorectal AC	1	1	28	0	0	1.00	1.00	1.00	1	-	-	-
Glioblastoma Multiforme	4	4	25	0	0	1.00	1.00	1.00	4	-	-	-

© 2019 Grewal JK et al. *JAMA Network Open*.

Brain Glioma	2	2	27	0	0	1.00	1.00	1.00	2	-	-	-
Liver Hepatocarcinoma	1	1	28	0	0	1.00	1.00	1.00	1	-	-	-
Pancreatic AC	2	2	27	0	0	1.00	1.00	1.00	2	-	-	-
Cutaneous Melanoma	1	1	28	0	0	1.00	1.00	1.00	1	-	-	-
Uterine Carcinosarcoma	1	1	28	0	0	1.00	1.00	1.00	1	-	-	-
Sarcoma	6	5	24	0	1	1.00	0.83	0.91	5	-	-	1
Lung AC	4	3	26	0	1	1.00	0.75	0.86	3	-	1	-
Mesothelioma	4	3	26	0	1	1.00	0.75	0.86	3	-	-	1
Lung SCC	1	1	28	1	0	0.50	1.00	0.67	1	-	-	-
UCEC	1	0	29	0	1	0.00	0.00	0.00	-	-	1	-
	**201**	**160**	**3128**	**19**	**41**	**0.80**	**0.76**	**0.75**	**160**	**7**	**13**	**21**

© 2019 Grewal JK et al. *JAMA Network Open*.

eFigure 1. Performance of Various Models (Distinct Based on Feature Selection and Architecture) on the Held-out Set.

The x-axis has been ordered by increasing class size, indicated in the first panel, and performance is shown on the CV-folds (black) and on the held-out set (yellow). As can be seen, the difference between CV-fold performance and held-out performance is large for small classes, but peters off as the class size approaches >100. When the classifier is augmented by addition of synthetic samples in the training folds (last panel), we see that there is an overall increase in performance for the rare classes, and the gap between mean-CV-precision and heldout precision is minimized. The line of best fit (loess) is indicated for each model, with standard error bounds in grey. The performance in different CV folds is shown by the black point (mean) with 1 standard deviation bars.

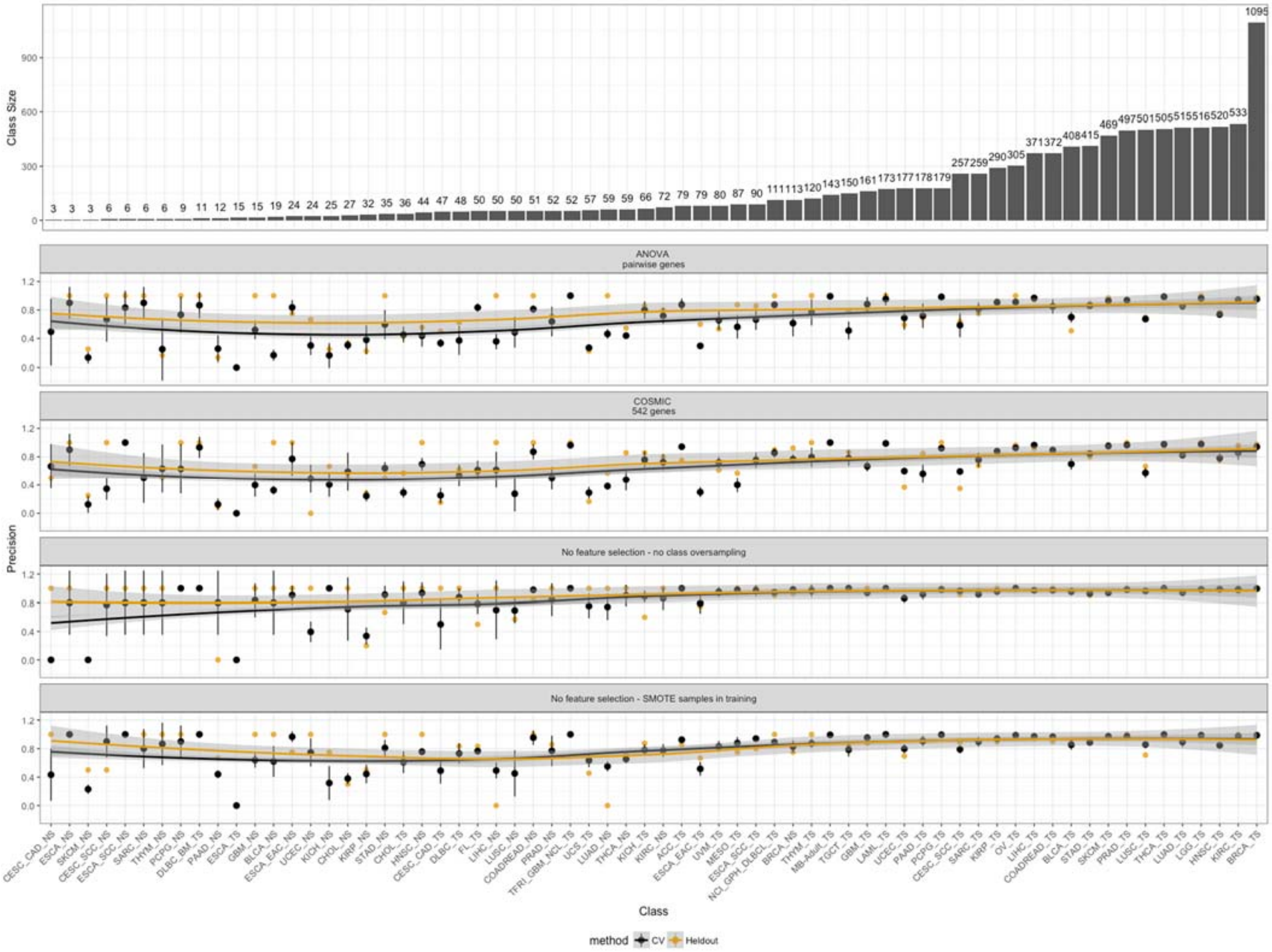

© 2019 Grewal JK et al. *JAMA Network Open*.

eFigure 2. Performance of Algorithms on CV Folds During Training**.**

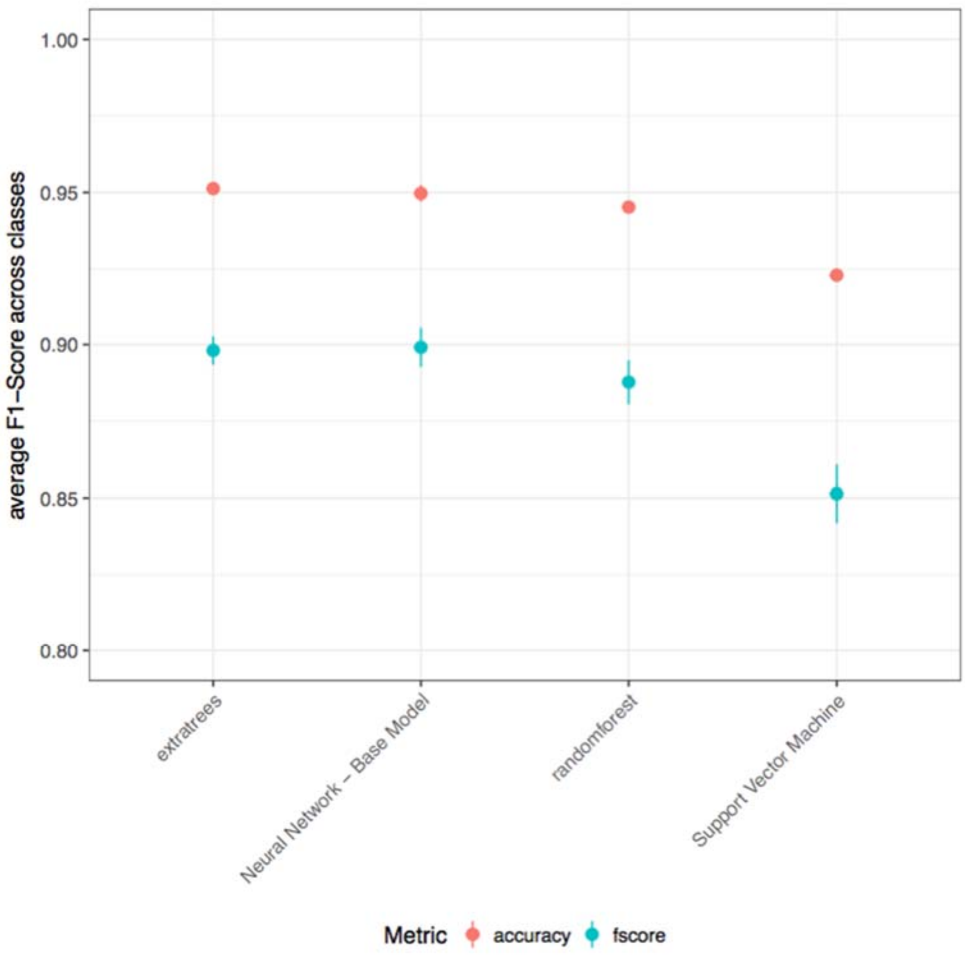

© 2019 Grewal JK et al. *JAMA Network Open*.

eFigure 3. Performance of SCOPE on the Held-out Set**.**

All 66 classes are shown, (a) showing performance between tumors and normal counterpart classes, and (b) showing cross-calling patterns originating from the normal class samples in the held-out set.

Performance of individual neural networks that make up SCOPE (n=5) are shown in grey points.

a) The average and 1 standard deviation spread of class-specific F1-score across these is shown in black (black point = mean, error bars = standard deviation). The two panels separate the normal (n=26) and tumor (n=40) tissue classes.

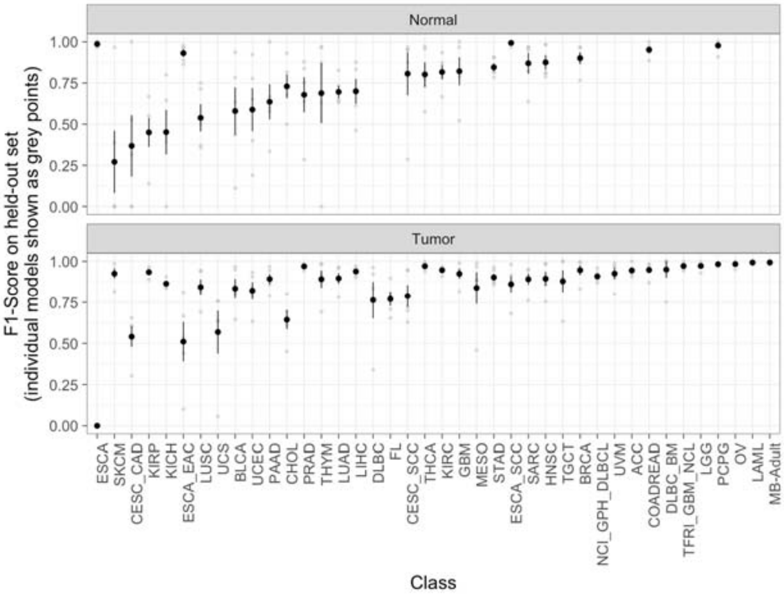

b) The average and 1 standard deviation spread of class-specific F1-scores across the ensemble machines is shown by the colored error bars. “_TS” and “_NS” indicate tumor and normal tissue classes respectively. Width of curves indicates proportion of cross-called samples from the originating class (direction indicated by arrow), with the smallest width corresponding to 15% of samples. As is evident, cross-calling is mostly between normal tissues from the same organ-system of origin.

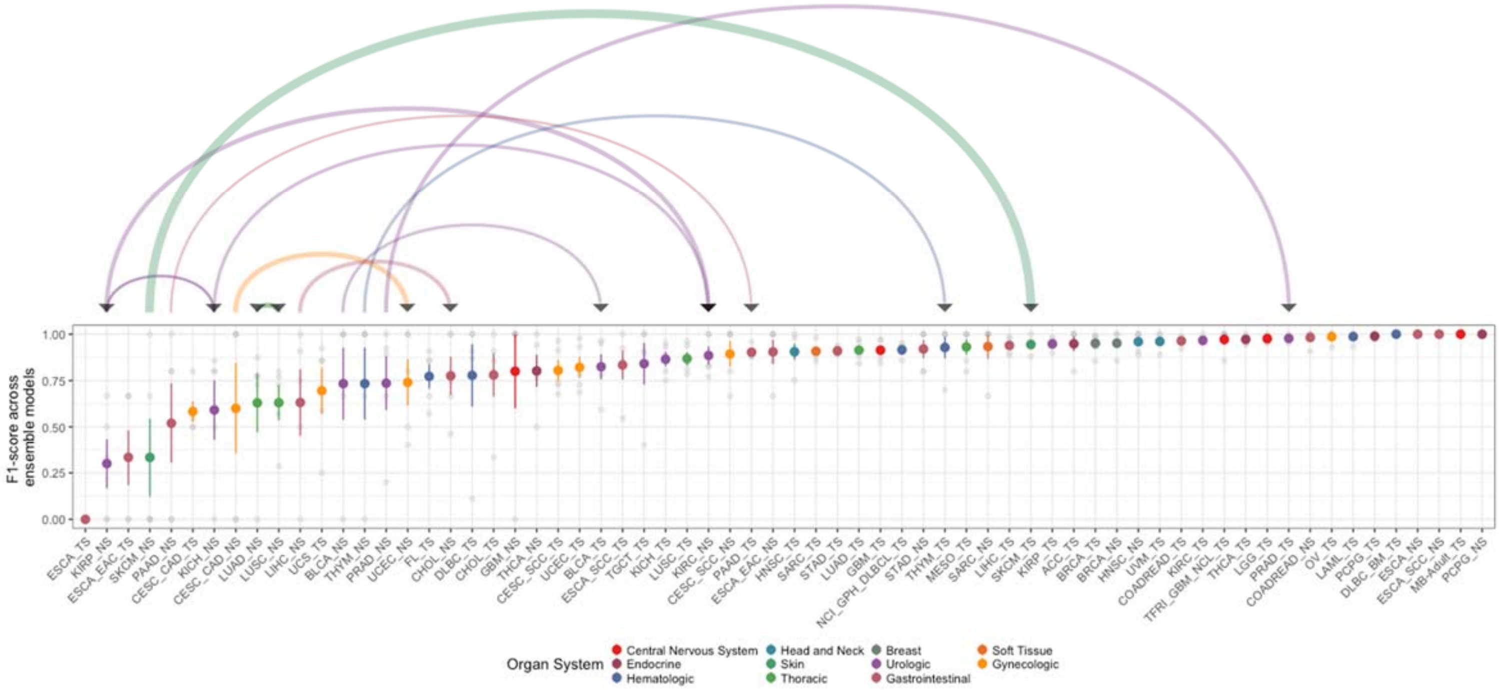

© 2019 Grewal JK et al. *JAMA Network Open*.

eFigure 4. The Performance of Individual Neural Networks on the Held-out Set

The SMOTE classifier is the 5th (bottom) panel. Class specific performances of each model, ordered by increasing class size, are shown. The names of the models are as defined in eTable 3. For the classes shown on the x-axis, _TS indicates a tumor class and _NS indicates a normal class. The performance of each model on each class is shown in grey points, and the line of best fit (loess) shown in blue with grey standard error bounds. Classes are ordered in increasing class size (class size shown in top panel).

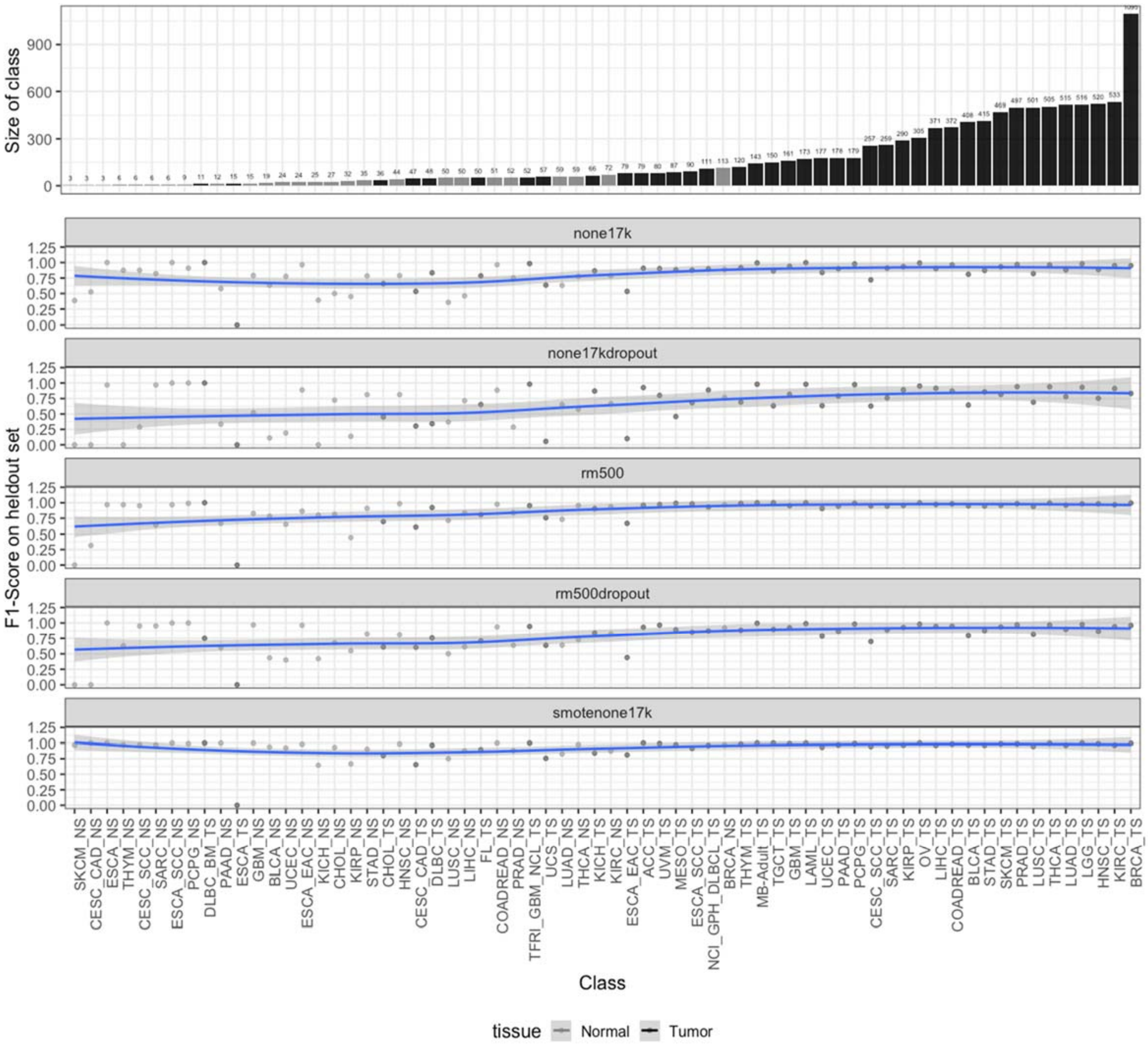

© 2019 Grewal JK et al. *JAMA Network Open*.

eFigure 5. t-SNE Plot of Transcriptomic Data in TCGA Training Cohorts**. All tumour types are shown.**

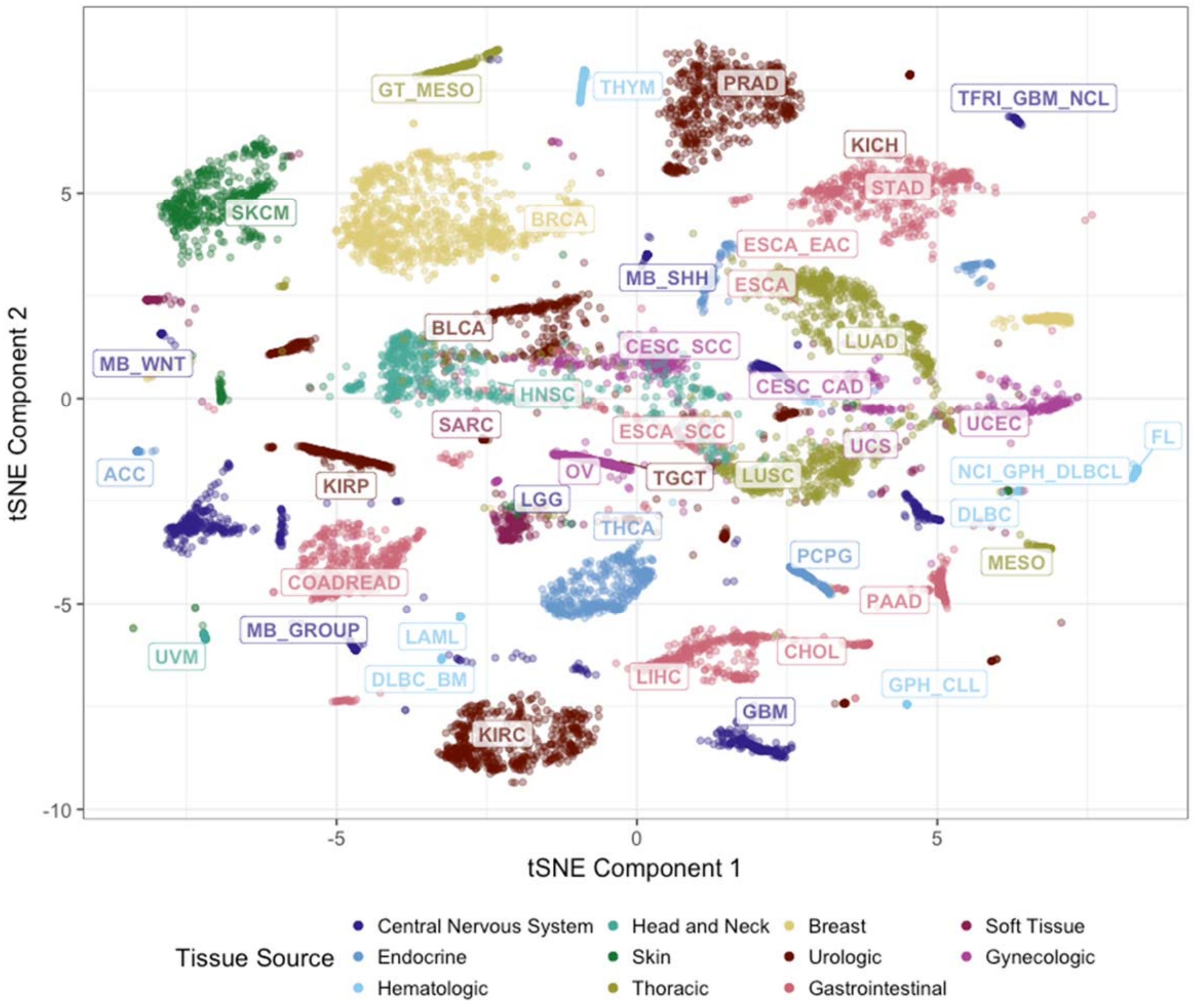

© 2019 Grewal JK et al. *JAMA Network Open*.

eFigure 6. t-SNE Plot of Transcriptomic Data in TCGA Training Cohorts**.**

Relevant gynecologic and gastrointestinal cancer types are shown.

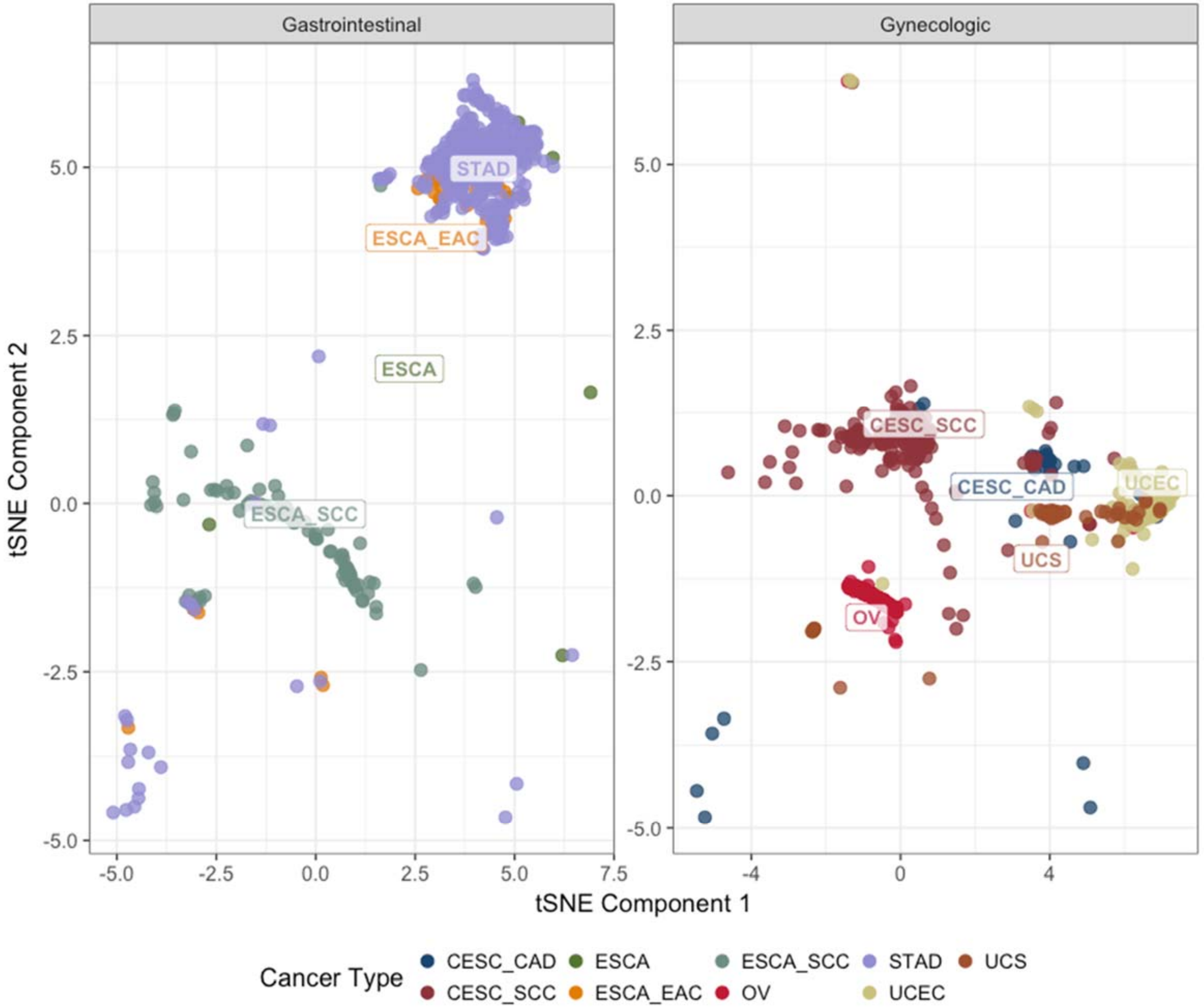

© 2019 Grewal JK et al. *JAMA Network Open*.

eFigure 7. A Detailed Version of Figure 2A, Whereby the Smaller Classes Are Shown Individually Instead of in Aggregate**.** AC = "Adenocarcinoma", CA = "Carcinoma", SCC = "Squamous Cell Carcinoma", CESC – AC = "Cervical/Endocervical Adenocarcinoma", UCEC= "Uterine Corpus Endometrial Carcinoma"

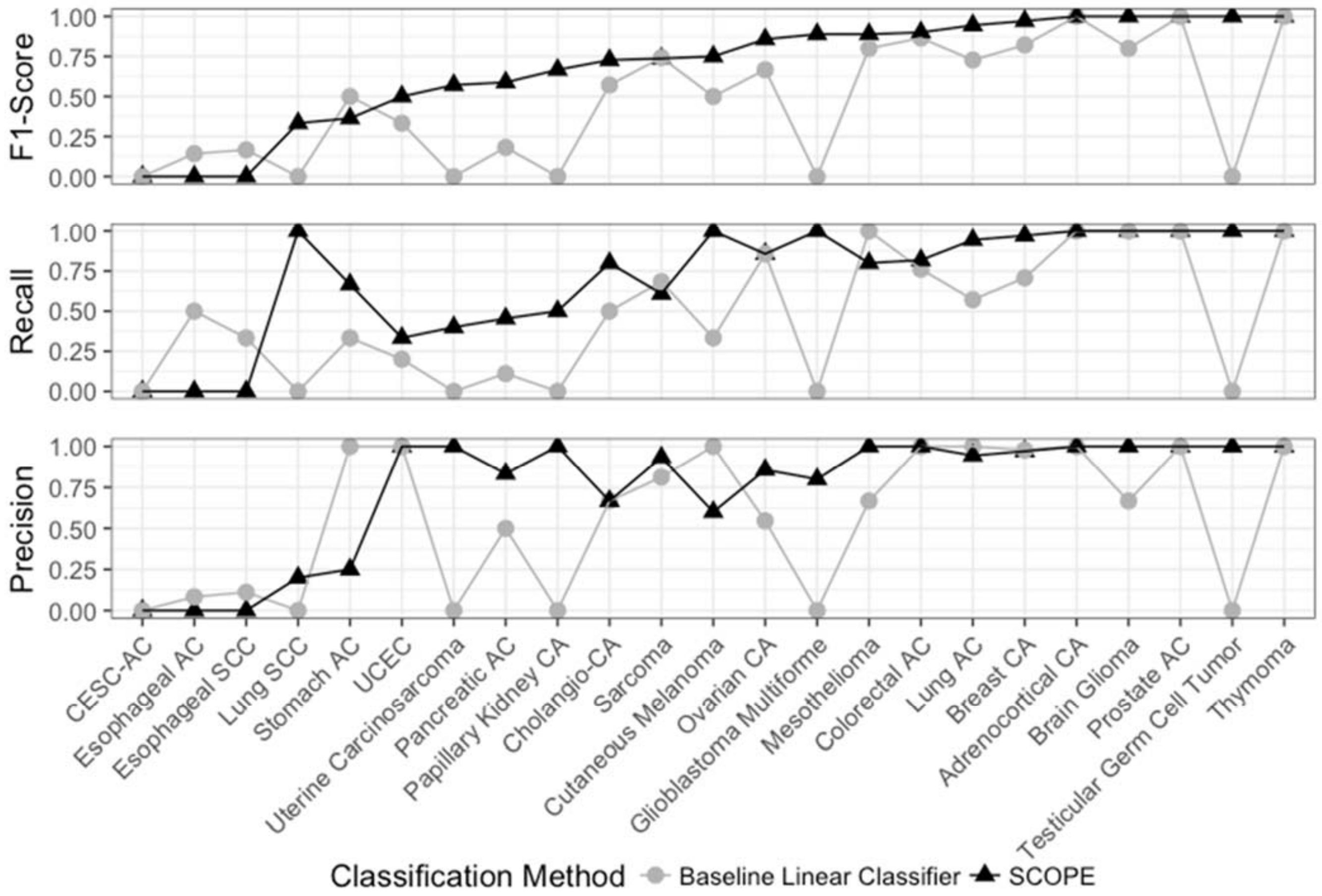

© 2019 Grewal JK et al. *JAMA Network Open*.

eFigure 8. The Distribution of Values for Tests of Association Between Classification Accuracy and a) Tumor Content (%), b) Confidence Score, and c) Training Class Size Are Shown.

A)

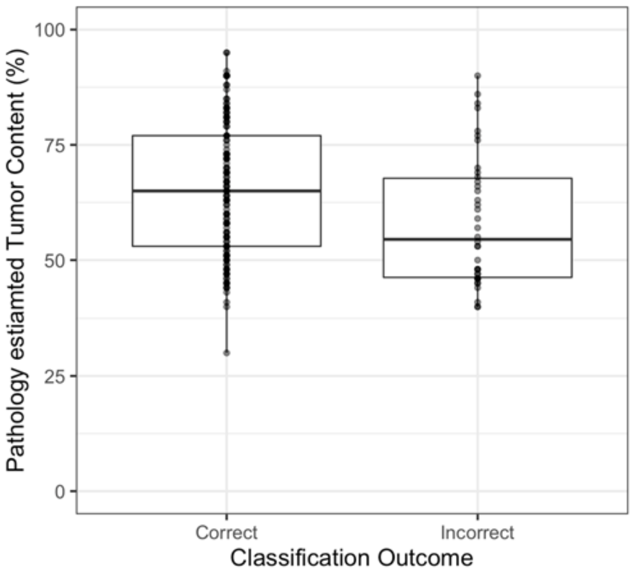

B)

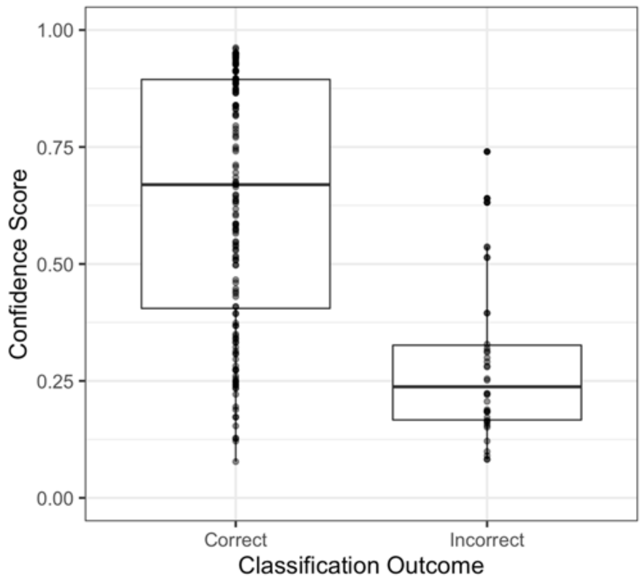

C)

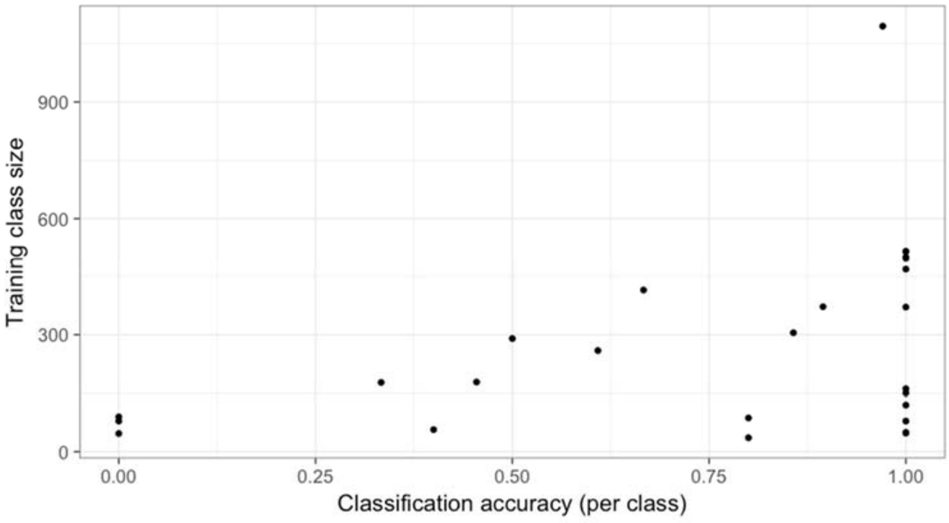

© 2019 Grewal JK et al. *JAMA Network Open*.

eFigure 9. Example Outputs From SCOPE in cases where the tested sample was (a) an epithelioid mesothelioma, correctly identified, (b) sarcomatoid mesothelioma, predicted with split confidence between mesothelioma and sarcoma, (c) a metastatic colorectal cancer, correctly identified, and (d), a metastatic stomach adenocarcinoma biopsied from the liver, incorrectly identified as the site of biopsy. The point colors indicate the Organ System of origin, as shown by the legend.

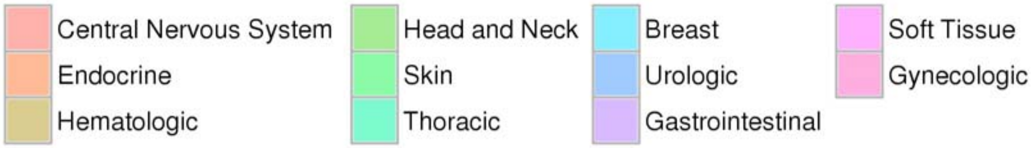

(a)

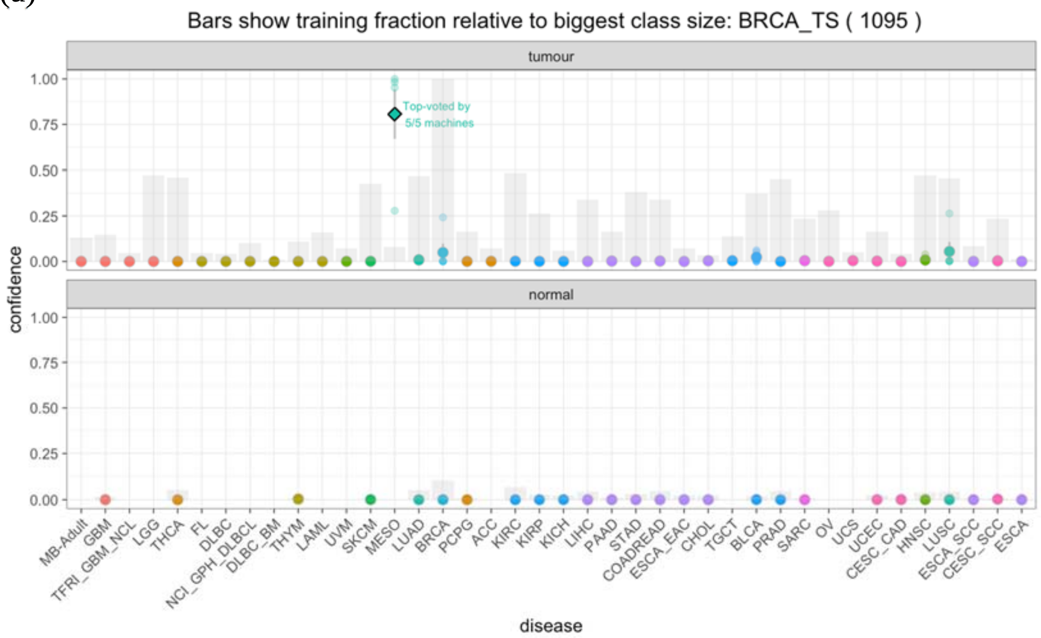

(b)

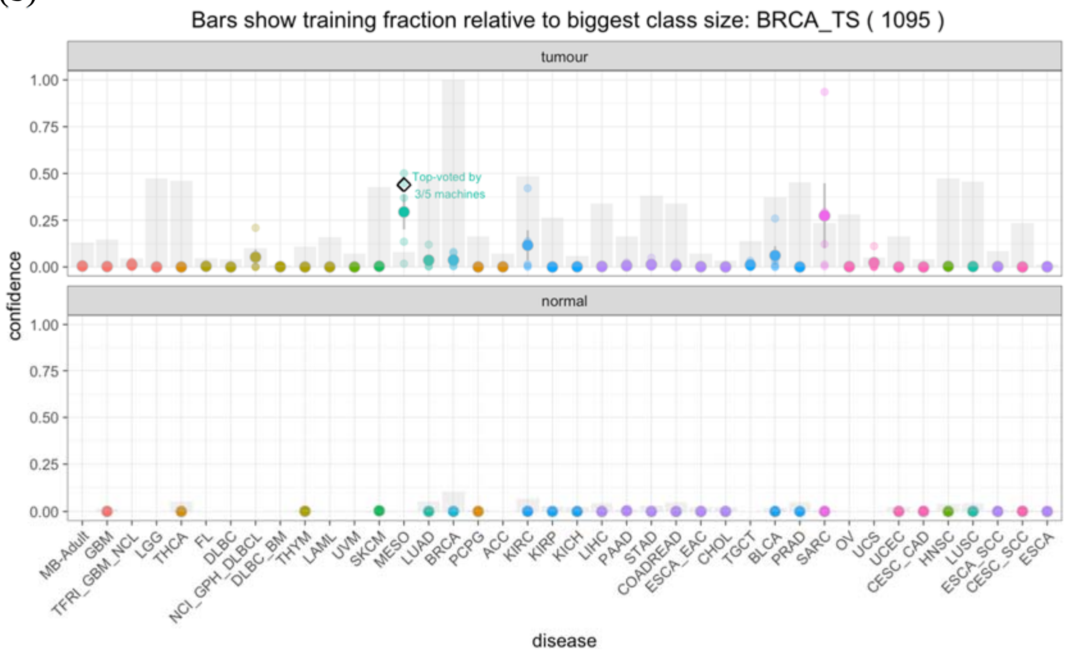

© 2019 Grewal JK et al. *JAMA Network Open*.

(c)

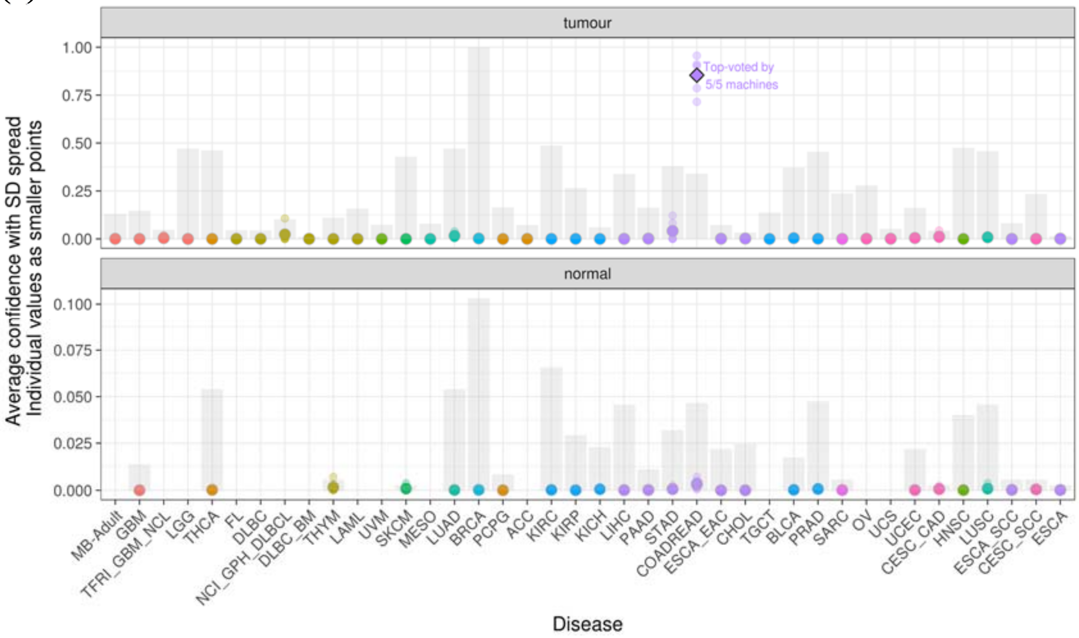

(d)

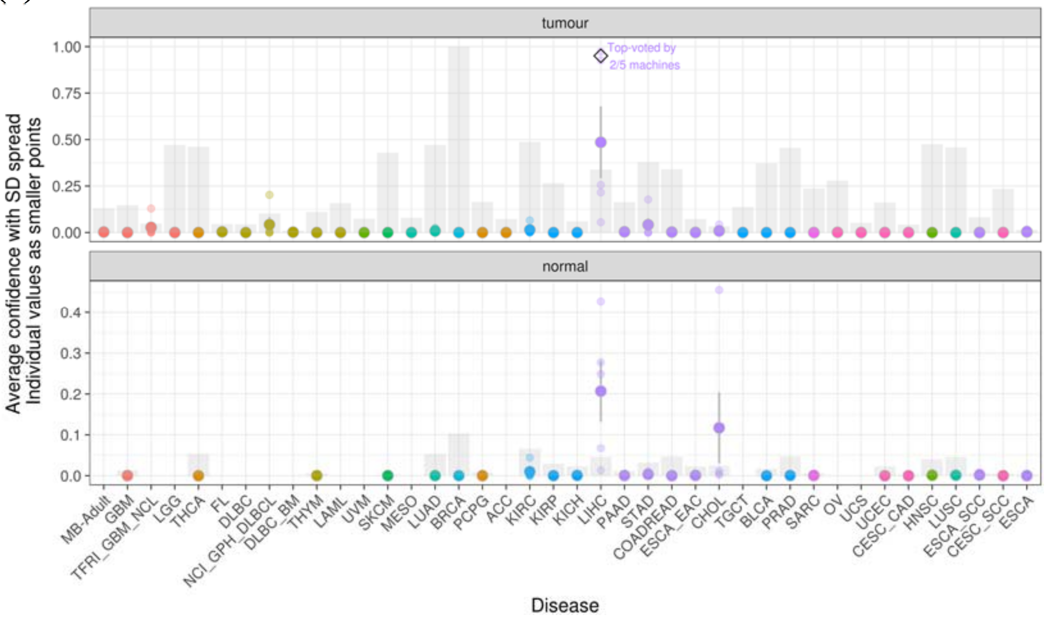

© 2019 Grewal JK et al. *JAMA Network Open*.

eReferences

[1] Morin RD, Johnson NA, Severson TM, et al. Somatic mutations altering EZH2 (Tyr641) in follicular and diffuse large B-cell lymphomas of germinal-center origin. Nat Genet. 2010;42(2):181-5.

[2] Pedregosa F, Varoquaux G. *Scikit-learn: Machine learning in Python*. Vol. 12. 2011, pp. 2825–2830. isbn: 9781783281930. doi: 10 . 1007 / s13398 - 014 - 0173 - 7 . 2. arXiv: arXiv : 1201.0490v2. url: http://dl.acm.org/citation.cfm?id=2078195.

[3] Bueno R, Stawiski EW, Goldstein LD, et al. Comprehensive genomic analysis of malignant pleural mesothelioma identifies recurrent mutations, gene fusions and splicing alterations. Nat Genet. 2016;48(4):407-16.

[4] Laskin J, Jones S, Aparicio S, et al. Lessons learned from the application of whole-genome analysis to the treatment of patients with advanced cancers. Cold Spring Harb Mol Case Stud. 2015;1(1):a000570.

[5] Chawla N.V., Bowyer K.W., Hall L.O., Kegelmeyer W.P. “SMOTE: Synthetic minority over-sampling technique”. In: *Journal of Arti- ficial Intelligence Research* 16 (2002), pp. 321–357. issn: 10769757. doi: 10.1613/jair.953. arXiv: 1106.1813.

[6] Dieleman S, Schülter J, Raffel C et al. *Lasagne: First release.* Aug. 2015. doi: 10.5281/zenodo.27878. url: http://dx.doi.org/10.5281/zenodo.27878.
